# Construction of an amylolytic *Saccharomyces cerevisiae* strain with high copies of α-amylase and glucoamylase genes integration for bioethanol production from sweet potato residue

**DOI:** 10.3389/fmicb.2024.1419293

**Published:** 2024-08-06

**Authors:** Xin Wang, Na Guo, Jingting Hu, Chenchen Gou, Xinyue Xie, Haobo Zheng, Aimei Liao, Jihong Huang, Ming Hui, Na Liu

**Affiliations:** ^1^National Engineering Research Center of Wheat and Corn Further Processing, Henan University of Technology, Zhengzhou, Henan, China; ^2^College of Biological Engineering, Henan University of Technology, Zhengzhou, Henan, China; ^3^School of Food and Pharmacy, Xuchang University, Xuchang, Henan, China; ^4^Collaborative Innovation Center of Functional Food by Green Manufacturing, Xuchang, Henan, China; ^5^Henan Provincial Engineering Laboratory of Preservation and Breeding of Industrial Microbial Strains, Zhengzhou, Henan, China; ^6^School of International Education, Henan University of Technology, Zhengzhou, Henan, China; ^7^State Key Laboratory of Crop Stress Adaptation and Improvement, College of Agriculture, Henan University, Kaifeng, Henan, China

**Keywords:** sweet potato residue fermentation, bioethanol production, recombinant amylolytic *Saccharomyces cerevisiae* strain construction, without exogenous enzyme addition, consolidated bioprocessing

## Abstract

Sweet potato residue (SPR) is the by-product of starch extraction from fresh sweet potatoes and is rich in carbohydrates, making it a suitable substrate for bioethanol production. An amylolytic industrial yeast strain with co-expressing α-amylase and glucoamylase genes would combine enzyme production, SPR hydrolysis, and glucose fermentation into a one-step process. This consolidated bioprocessing (CBP) shows great application potential in the economic production of bioethanol. In this study, a convenient heterologous gene integration method was developed. Eight copies of a *Talaromyces emersonii* α-amylase expression cassette and eight copies of a *Saccharomycopsis fibuligera* glucoamylase expression cassette were integrated into the genome of industrial diploid *Saccharomyces cerevisiae* strain 1974. The resulting recombinant strains exhibited clear transparent zones in the iodine starch plates, and SDS-PAGE analysis indicated that α-amylase and glucoamylase were secreted into the culture medium. Enzymatic activity analysis demonstrated that the optimal temperature for α-amylase and glucoamylase was 60–70°C, and the pH optima for α-amylase and glucoamylase was 4.0 and 5.0, respectively. Initially, soluble corn starch with a concentration of 100 g/L was initially used to evaluate the ethanol production capability of recombinant amylolytic *S. cerevisiae* strains. After 7 days of CBP fermentation, the α-amylase-expressing strain 1974-temA and the glucoamylase-expressing strain 1974-GA produced 33.03 and 28.37 g/L ethanol, respectively. However, the 1974-GA-temA strain, which expressed α-amylase and glucoamylase, produced 42.22 g/L ethanol, corresponding to 70.59% of the theoretical yield. Subsequently, fermentation was conducted using the amylolytic strain 1974-GA-temA without the addition of exogenous α-amylase and glucoamylase, which resulted in the production of 32.15 g/L ethanol with an ethanol yield of 0.30 g/g. The addition of 20% glucoamylase (60 U/g SPR) increased ethanol concentration to 50.55 g/L, corresponding to a theoretical yield of 93.23%, which was comparable to the ethanol production observed with the addition of 100% α-amylase and glucoamylase. The recombinant amylolytic strains constructed in this study will facilitate the advancement of CBP fermentation of SPR for the production of bioethanol.

## 1 Introduction

Sweet potato is a high-yielding and adaptable food crop, which is widely cultivated in tropical and subtropical regions (Karmee, 2016). The annual output of sweet potatoes can reach approximately 100 million tons, with China accounting for the majority of global production, at approximately 80% (Xia et al., 2019). At present, the sweet potato–processing industry focuses on starch extraction, food production, and microbial fermentation (El Sheikha and Ray, 2016). Sweet potato residue (SPR), which is extracted from fresh sweet potatoes after crushing, washing, and filtering, is primarily composed of starch, cellulose, and pectin (Gou et al., 2023). However, given its low water solubility and high levels of polysaccharides and proteins, SPR is susceptible to mildew and contamination. Rancid SPR releases methane as a residue, which is the second most prevalent greenhouse gas and whose impact on climate change can be more than 25 times that of carbon dioxide (Lu et al., 2013). In general, SPR is used in animal protein feed production, pectin extraction, and dietary fiber extraction (Aziz and Mohsen, 2002; Hao et al., 2014; Pagana et al., 2014; Wang et al., 2016). However, long-term storage of SPR can result in mycotoxin production, which limits its application as animal feed. Meanwhile, the extraction rates for pectin and dietary fibers from SPR are insufficiently high to reach the industrial production scale. Researchers have developed biotechnologies to transform SPR into various biological products through microbial fermentation. This process involves the hydrolysis of starch and cellulose by enzymes, thereby producing monosaccharides and oligosaccharides, which can be fermented to produce butanol and other products (Jin et al., 2022).

Bioethanol is widely regarded as a potential source of energy and an alternative to fossil fuels (Osman et al., 2021). At present, bioethanol is predominantly produced from sugar and starchy food crops, such as sugar cane, corn, and cassava, because of the well-established processing techniques and high ethanol yields associated with these crops (Cripwell et al., 2020). Nevertheless, the development of the corn ethanol industry is constrained by the rising demand for food and animal feed. Furthermore, feedstock represents an important proportion of the overall production costs of bioethanol, necessitating the identification of alternative, low-cost feedstocks (Wang et al., 2019). SPR, a cheap and abundant starchy by-product from the sweet potato–processing industry, shows great application potential in this regard.

Irrespective of the feedstock, there are other substantial costs associated with the current starch-to-ethanol production process (Görgens et al., 2014). These costs are predominantly associated with conventional energy-intensive gelatinization or the addition of exogenous enzyme cocktails for the liquefaction and saccharification of raw starch. The energy requirement for conventional gelatinization accounts for 10%–20% of the fuel value of ethanol in a typical refinery (Lorenzo and Marina, 2010). Moreover, the cost of enzymes represents 8% of the total processing cost (Görgens et al., 2014). Therefore, the production of ethanol from starch can benefit from the combination of all these steps into a single process, which is known as consolidated bioprocessing (CBP). In such a process, genetically engineered ethanologenic yeast strains, such as amylolytic *Saccharomyces cerevisiae* strains, are required to produce raw starch-degrading enzymes that enable the yeast to simultaneously hydrolyze the starch and ferment the resulting sugars to ethanol (Chandel et al., 2018; Cripwell et al., 2019b).

A number of *S. cerevisiae* strains with raw starch CBP capabilities have been developed with varying degrees of success (Viktor et al., 2013; Favaro et al., 2015; Sakwa et al., 2018). Most previous studies have focused on the engineering of *S. cerevisiae* laboratory strains (Cripwell et al., 2019a), which are easy to manipulate and allow for the screening of transformants without antibiotic selection. However, laboratory strains showed lower thermotolerance and decreased glucose fermentation rates when compared with industrial yeast strains (Kong et al., 2018). Furthermore, starch-degrading enzymes genes were always expressed using episomal plasmids rather than integration into the genome, and the selection marker and copy number of the plasmids will affect the growth ability and cellular metabolism of the cell (Karim et al., 2013). Given the aforementioned considerations, Cripwell et al. used the industrial *S. cerevisiae* yeast strain Ethanol Red as a host, integrating seven copies of the codon-optimized *Talaromyces emersonii* glucoamylase-encoding gene (*temG_Opt*) and four copies of the native *T. emersonii* α-amylase-encoding gene (*temA*) into the genome via δ-integration. The recombinant industrial amylolytic yeast strain could produce 89.35 g/L ethanol from 200 g/L raw corn starch in a single step after 192 h at 30°C, which represents the highest value to date (Cripwell et al., 2019a).

In this study, the industrial bioethanol-producing diploid *S. cerevisiae* 1974 was selected as the parental strain. The *T. emersonii* α-amylase gene (*temA*) and codon-optimized *Saccharomycopsis fibuligera* glucoamylase gene (*GA*), both of which have been proven to have a high starch-hydrolyzing activity were integrated into the specific sites on the *S. cerevisiae* 1974 genome (Cripwell et al., 2019a; Wang et al., 2021). Based on a quick and easy high-copy number gene integration method that was developed, a recombinant *S. cerevisiae* strain harboring eight copies of *temA* and eight copies of *GA* was constructed. The recombinant yeast strains were assessed for their starch hydrolysis capability using iodine starch plates. In addition, SDS-PAGE analysis was performed to analyze the expression of recombinant proteins in the supernatant fermentation broth. The effect of time, pH, and temperature on the starch-hydrolyzing enzymatic activity was also investigated. Finally, the ability of recombinant strains to ferment soluble starch and SPR to produce ethanol was also evaluated. To the best of our knowledge, this study is the first to utilize a starch-hydrolyzing yeast strain for CBP fermentation of SPR to produce ethanol.

## 2 Materials and methods

### 2.1 Strains, plasmids, and growth conditions

The strains and plasmids used in this study are listed in Table 1. In brief, the industrial bioethanol production diploid *S. cerevisiae* strain 1974 was used as the original host. All molecular cloning operations were conducted in *Escherichia coli* strain DH5α. In addition, strain DH5α was cultured in LB medium at 37°C, and 100 μg/mL ampicillin was added if necessary. For routine *S. cerevisiae* molecular engineering, strains were maintained in a YPD medium. Antibiotics (300 μg/mL G418 or 400 μg/mL hygromycin) was added as required. The composition of the medium used is shown in the supporting information. α-Amylase (Beijing Aoboxing Biotechnology Co., Ltd, 3700 U/g) and glucoamylase (Beijing Aoboxing Biotechnology Co., Ltd,100000 U/g) were used in the CBP fermentation test.

**TABLE 1:** Plasmids and strains used in this study.

Strains	Characteristics	Source
1974	An industrial diploid ethanol producing *S. cerevisiae* strain	Wang et al., 2019
1974-GA	1974 XII-5::4 copies of *GA*, X-2::4 copies of *GA*.	This study
1974-temA	1974 X-3::4 copies of *temA*, XI-2::4 copies of *temA*.	This study
1974-GA-temA	1974 XII-5::4 copies of *GA*, X-2::4 copies of *GA*, X-3::4 copies of *temA*, XI-2::4 copies of *temA*.	This study
**Plasmids**		
pYIE2-2GA-δ	Codon-optimized *S. fibuligera* glucoamylase expression gene harboring plasmid, which is used as PCR template in this study.	Wang et al., 2021
ptemA	*T. emersonii* αα-amylase expression gene harboring plasmid, which is used as PCR template in this study.	This study
pX-2-2GA	X-2 site *GA* integration plasmid, TEF1p-GA-ADH1t, GAPp-GA-CYC1t.	This study
pXII-5-2GA	XII-5 site *GA* integration plasmid, TEF1p-GA-ADH1t, GAPp-GA-CYC1t.	This study
pX-3-2temA	X-3 site *temA* integration plasmid, TEF1p-temA-ADH1t, GAPp-temA-CYC1t.	This study
pXI-2-2temA	XI-2 site *temA* integration plasmid, TEF1p-temA-ADH1t, GAPp-temA-CYC1t.	This study

### 2.2 Plasmid and strain construction

The KOD-plus-neo DNA polymerase (Toyobo, Japan) or KOD FX polymerase (Toyobo, Japan) was used for PCR amplifications. The DNA restriction enzymes for cloning were obtained from Thermo Fisher Scientific (USA). The primers used for plasmid construction are listed in Supplementary Table 1. The codon-optimized *S. fibuligera* glucoamylase gene (GenBank accession number: MW082635) was amplified from the plasmid pYIE2-2GA-δ. The *T. emersonii* amylase gene *temA* was synthesized by GenScript Biotech Corporation (China), and the resulting plasmid was ptemA. Detailed procedures for plasmid and strain construction are described in the supporting information. The map of the plasmids pX-2-2GA, pXII-5-2GA, pX-3-2temA, and pXI-2-2 are shown in Supplementary Figures 1, 2.

### 2.3 Determination of the copy number of GA and temA genes in recombinant S. cerevisiae strain by colony PCR and real-time PCR

After the transformation of *GA* or *temA* gene expression cassette fragments, several transformants grown in the YPD-G418-HYG plate were selected randomly and pretreated with lysis buffer for colony PCR. One primer was located within the *GA* or *temA* gene expression cassette, while another primer was located in the chromosomal homology arm. This combination of primers was used to ascertain whether the target genes had been integrated into the specific region of the chromosome. Genomic DNA was extracted from the positive transformants and used as the next PCR template. In addition, the primer pairs located in the up and down regions of the heterologous gene insertion sites were selected, and the strain 1974 was used as a control. If a short fragment was amplified, then only one copy of the chromosome had been inserted by heterologous genes. By contrast, no fragment was amplified if the two chromosomes had been inserted.

For analysis of the gene copy number, the strains’ genomic DNA was isolated with a TIANamp Yeast DNA Kit (Tiangen Biotech [Beijing] Co., Ltd) according to the manufacturer’s protocol and quantified by NanoDrop ND-1000 (Thermo Fisher Scientific). The fold changes of the gene copy number compared with the reference gene ACT1 were determined using the equation, *N*_Target_ */ N*_Reference_ = (2^Ct (Reference)^ /2^Ct (Target)^) × 2. Note that the copy number of *ACT1* is 2 because the strain is a diploid yeast. The primers used for real-time quantitative PCR are listed in Supporting Information Supplementary Table 1.

### 2.4 Starch-hydrolyzing plate and SDS-PAGE analysis

The recombinant *S. cerevisiae* strains were inoculated into 3 mL of YPD medium and cultured overnight at 30°C and 240 rpm until reaching an OD_600_ value of 4.7. Subsequently, 10 μL of the culture supernatant was inoculated into the YPS plate and incubated at 30°C for 72 h. After incubation, the starch-degradation test plate was flooded with approximately 10 mL of 50 mM potassium iodide–iodine solution and incubated for 15 min at room temperature. The excess iodine solution was poured off, and the plates were washed with 1 M NaCl. The formation of a clear hydrolysis zone around the recombinant strains indicated the presence of starch-hydrolyzing enzymatic activity.

The protein content of aliquots (100 μL) of the medium was precipitated with 15% TCA and left on ice for 30 min. Subsequently, centrifugation in a microcentrifuge (10,600 × g, 15 min, 4°C) was performed to remove the supernatants of the TCA-treated samples, after which the pellets were washed with ice-cold acetone. Following a second round of centrifugation in a microcentrifuge (10,600 × g, 15 min, 4°C), the supernatants were discarded, and the pellets were air dried. Then, SDS-PAGE sample buffer was added, and the proteins were separated on 15% SDS-PAGE gels.

### 2.5 Genetic stability and enzymatic activity analysis

The frozen 1974-GA-temA glycerol stock was streaked onto a YPD plate and cultured at 30°C. Then, the single colony was streaked onto a new YPD plate. This procedure was repeated until 30 serial passages were performed. Colonies from the 10th, 20th, and 30th passages were selected to test the starch hydrolysis activity using the iodine vapor method.

For quantitative amylase analysis, single clones or frozen glycerol stock cultures of 1974, 1974-GA, and 1974-GA-temA were inoculated into 3 mL of YPD medium in test tubes and grown overnight at 30°C and 240 rpm. A total of 200 μL of the cultures were transferred to 50 mL of fresh YPD medium. After 72 h of incubation, the cultures were centrifuged at 12,000 rpm for 10 min. The resulting supernatant was utilized for the analysis of starch-hydrolyzing enzymatic activity. The reaction mixture, comprising 450 μL of 1% soluble starch in 0.1 M citrate–phosphate buffer (pH 4.5) and 50 μL of enzyme solution, was incubated at 40°C for 30 min. Then, the reaction was terminated by heating for 10 min in boiling water. A control sample was incubated for 10 min in boiling water to inactivate the enzyme and incubated under the same conditions. The reducing sugar content was measured using the dinitrosalicylic acid method (Gou et al., 2023). One unit of enzymatic activity was defined as the amount of enzyme that produced 1 μmol of reducing sugar (equivalent to 1 μmol glucose) per minute under the abovementioned standard assay conditions. A standard curve of the glucose is shown in Supplementary Figure 4.

### 2.6 Fermentation studies

For soluble starch fermentation, the recombinant strains were inoculated into 6 mL of YPD medium in test tubes and grown overnight at 30°C and 240 rpm, the OD_600_ of the strain was about 6.0. The cells were harvested by centrifugation at 12,000 rpm, washed two times with sterile water, suspended using YPS medium and inoculated into 50 mL of YPS medium in anaerobic flasks at an initial inoculation volume of 10% (v/v). During fermentation, samples were taken at specific time intervals for OD_600_ readings and metabolite analysis.

SPR was provided by Luoyang Feed Factory of Henan Dongfang Zhengda Co., Ltd., China. The dried SPR was pulverized, passed through a 60-mesh sieve, and stored at room temperature. The dried SPR is composed of 51.94% ± 0.55% starch, 18.22% ± 0.47% cellulose, 3.38% ± 0.02% hemicellulose, 1.66% ± 1.31% pectin, 2.92% ± 0.03% protein, and 2.23% ± 0.01% ash (Gou et al., 2023). For SPR fermentation, the recombinant *S. cerevisiae* strain 1974-GA-temA was inoculated into 3 mL of YPD medium in test tubes and grown overnight at 30°C and 240 rpm. A total of 200 μL of the cultures was transferred to 25 mL of YPD medium in flasks to repeat the procedure. After aerobic growth, cells were harvested by centrifugation at 12,000 rpm, washed two times with sterile water, and inoculated into the SPR medium in a 250 mL anaerobic flask at an inoculation volume of 10% (v/v). The SPR medium was prepared by weighing 10 g of SPR powder and mixing it with deionized water in a 1:6 solid-to-liquid ratio. The pH was set to 5.0 with 1M HCl or 1 M NaOH. For α-amylase and glucoamylase addition test, a total of 0.13 g of α-amylase (48 U/g SPR) and 0.03 g of glucoamylase (300 U/g SPR) were used to supplement the CBP fermentation of strains, which were designated as 100% dosage. Samples were taken at specific time intervals for OD_600_ readings and metabolite analysis during fermentation. The OD_600_ values, as well as glucose and ethanol concentrations, were determined in accordance with a previous report (Gou et al., 2023).

## 3 Results and discussion

### 3.1 High copy number of α-amylase and glucoamylase integration strain construction

**FIGURE 1:**
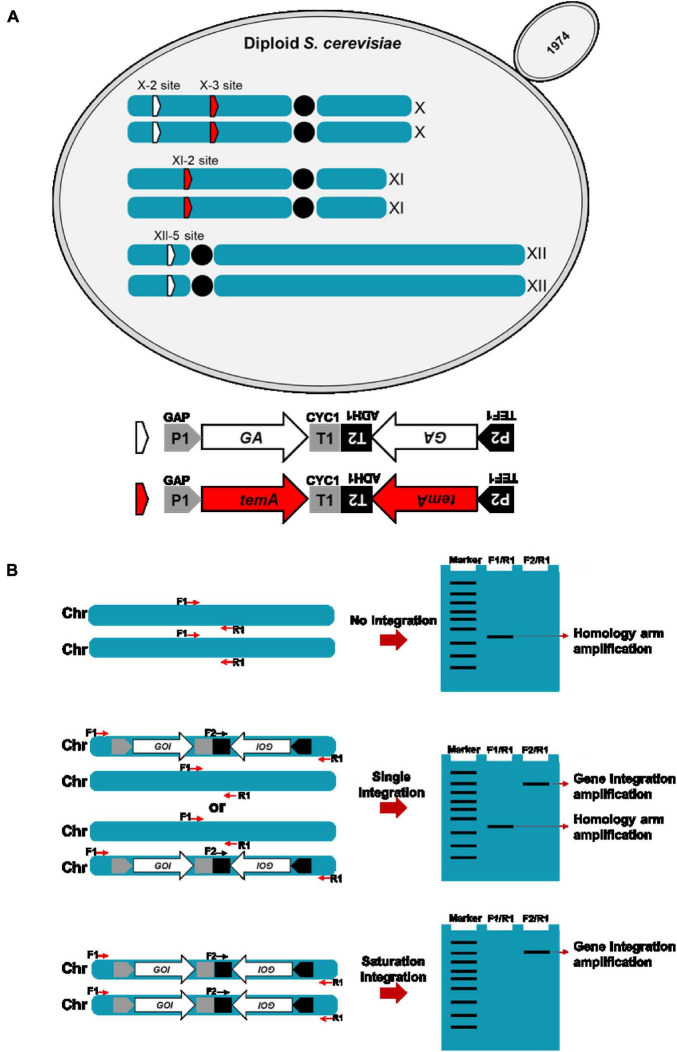
Schematic representation of the construction of the αα-amylase- and glucoamylase-producing *S. cerevisiae* strain **(A)** and gene copy number verification by PCR **(B)**. **(A)** The *GA* expression cassettes were integrated at chromosome XII-5 and X-2 sites. *temA* expression cassettes were integrated at chromosome X-3 and XI-2 sites. P1, GAP promoter. T1, CYC1 terminator. P2, TEF1 promoter. T2, ADH1 terminator. **(B)** Sketch map of gene copy number determination by using conventional PCR.

In this study, the industrial diploid *S. cerevisiae* strain 1974 was selected as the expression host due to its excellent fermentation capacity in the bioethanol production field (Wang et al., 2021). Two copies of the codon-optimized *S*. *fibuligera* glucoamylase expression gene were cloned into plasmids pX-2-2GA and plasmid pXII-5-2GA, respectively. Two copies of the *T. emersonii* α-amylase expression gene were also cloned into the plasmids pX-3-2temA and pXI-2-2temA. *GA* and *temA* genes were expressed under the control of the strong promoter and terminator combinations *P*_*ENO1*_-*T*_*ENO1*_ and *P*_*ADH1*_-*T*_*PDH1*_. In preventing the potential loss of the *GA* and *temA* gene pairs during mitotic recombination, the two copies were assembled in a tail-to-tail manner (Figure 1A). The *Not*I-linearized *GA* expression cassette was integrated into the chromosome XII-5 and X-2 sites of the 1974 genome and verified using diagnostic PCR. In parallel, the *Not*I-linearized *temA* expression cassette was integrated into the chromosome X-3 and XI-2 sites of the 1974 genome and verified using diagnostic PCR (Figure 1B).

To verify the integration of foreign genes, two pairs of primers were designed for PCR. One pair is located on the homology arms surrounding the integration site (F1/R1), whereas the other pair is located within the integrated gene and downstream homology arms (F2/R1, Figure 1B). Initially, the F2/R1 primer pair is used to determine whether the foreign gene is integrated at a specific site. The presence of a band indicates that the foreign gene expression cassette has been integrated into at least one chromosome. Subsequently, the F1/R1 primers are used to confirm these transformants. If a short fragment (homologous arm region) is amplified, then only one chromosome has integrated the foreign gene expression cassette (single integration). Conversely, the absence of the amplification of a short fragment indicates that both chromosomes have integrated the foreign gene expression cassette (saturation integration), resulting in four copies (Figure 1B).

**FIGURE 2:**
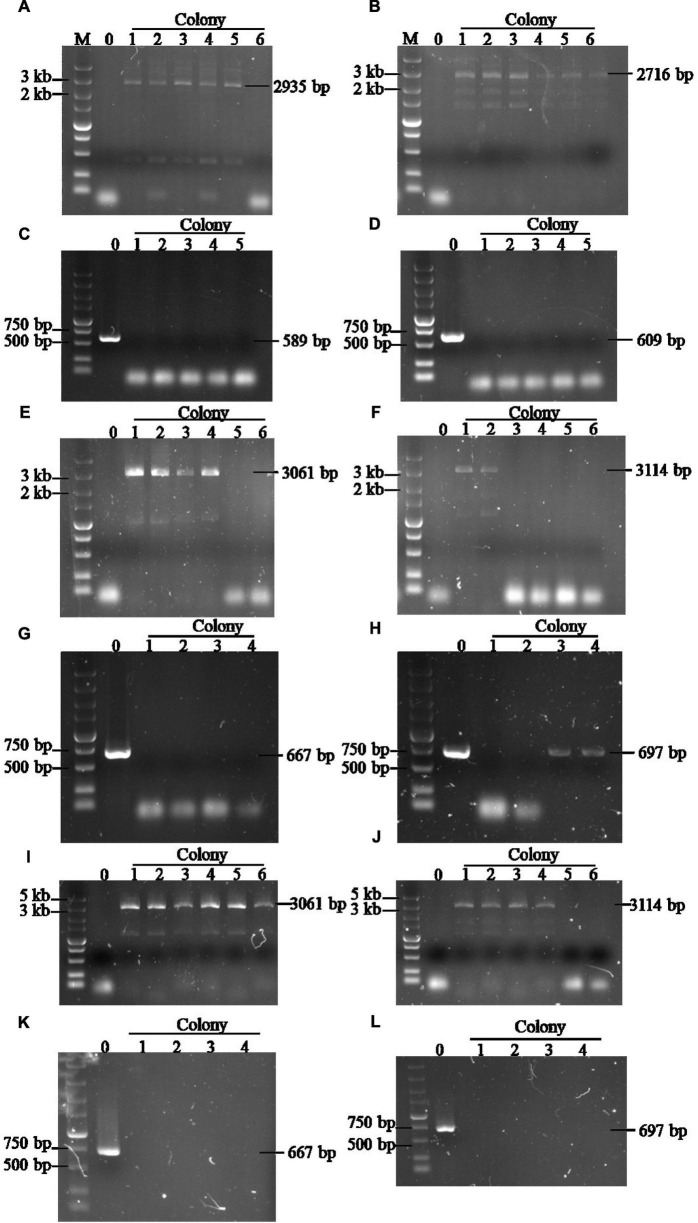
Agarose gel electrophoretic analysis of the *GA* copy number in strain 1974-GA **(A–D)**, *temA* copy number in strain 1974-temA **(E–H)**, and *GA* and *temA* copy number in strain 1974-GA-temA **(I–L)**. **(A,B)** Verification of *GA* integration at X-2 and XII-5 sites using F2/R1 primers (based on 1974 strain). **(C,D)** Verification of *GA* integration at X-2 and XII-5 sites using F1/R1 primers, respectively (based on 1974 strain). **(E,F)** Verification of *temA* integration at X-3 and XI-2 sites using F2/R1 primers, respectively (based on 1974 strain). **(G,H)** Verification of *temA* integration at X-3 and XI-2 sites using F1/R1 primers, respectively (based on 1974 strain). **(I,J)** Verification of *temA* integration at X-3 and XI-2 sites using F2/R1 primers, respectively (based on 1974-GA strain). **(K,L)** Verification of *temA* integration at X-3 and XI-2 sites using F1/R1 primers, respectively (based on 1974-GA strain). Number zero refers to the control strain 1974. The original gel of Figures 2A, B was presented as Supplementary Figure 5. The original gel of Figure 2C was presented as Supplementary Figure 6. The original gel of Figure 2D was presented as Supplementary Figure 7. The original gel of Figures 2E, F was presented as Supplementary Figure 8. The original gel of Figure 2G was presented as Supplementary Figure 9. The original gel of Figure 2H was presented as Supplementary Figure 10. The original gel of Figures 2I, J was presented as Supplementary Figure 11. The original gel of Figure 2K was presented as Supplementary Figure 12. The original gel of Figure 2L was presented as Supplementary Figure 13.

A high expression level of exogenous genes in *S. cerevisiae* is often necessary to overexpress pathway enzymes leading to bioethanol production (Myburgh et al., 2019; Wang et al., 2019). However, the common methods used for identifying gene copy numbers, such as real-time PCR or genome sequencing, are time consuming and laborious. Based on the site-specific gene copy number identification method developed in this study, several transformants were selected randomly to determine the gene copy number after *GA* and *temA* expression cassette transformation. Figures 2A, B demonstrate that the *GA* expression cassette was successfully integrated at the X-2 and XII-5 sites in colony 1 to colony 5 except for colony 6, which was integrated only at the XII-5 site. No band was amplified using the primer pairs F1/R1 (Figures 2C, D), indicating that both chromosomes have integrated the *GA* expression cassette, resulting in eight copies. Using the same method, six colonies were randomly selected to verify the integration of the *temA* expression cassette (Figures 2E–H). Colonies 1 and 2 exhibited saturation integration for the *temA* expression cassette. To construct stain 1974-GA-temA, the temA expression cassette was transformed into strain 1974-GA (Supplementary Figure 3), and strain-harbored newly integrated eight copies of the *temA* expression cassette were selected (Figures 2I–L). Copy numbers for integrated genes in each genome were also determined by using real-time PCR. As shown in Table 2, *GA* copy number in strain 1974-GA was 7.75, *temA* copy number in strain 1974-temA was 7.78, *GA* and *temA* copy number in strain 1974-GA-temA were 8.02 and 8.43, respectively. The copy number determined by real-time PCR was consistent with the colony PCR method.

**TABLE 2:** Genes copy number determination by real-time PCR.

Genes	1974-GA	1974-temA	1974-GA-temA
*GA*	7.75 ± 0.19		8.02 ± 0.31
*temA*		7.78 ± 0.23	8.43 ± 1.16

The values are the averages calculated from C_t_ values measured in triplicate reactions with recombinant strains’ DNA.

### 3.2 Functional secretion of recombinant α-amylase and glucoamylase in recombinant S. cerevisiae strains

**FIGURE 3:**
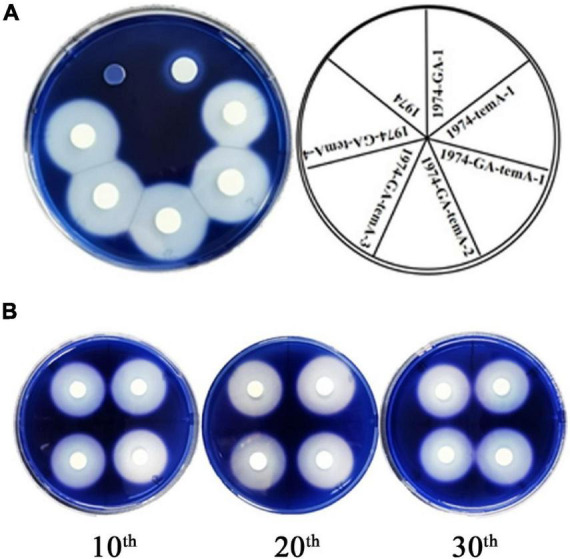
Hydrolysis zones indicate the production of starch-degrading enzymes **(A)** and genetic stability evolution **(B)**.

Following the cultivation of recombinant *S. cerevisiae* strains in YPS medium for 3 days, iodine staining was conducted using iodine particles (Cripwell et al., 2019b). The results demonstrated that no transparent circle was formed around the control strain 1974, whereas a transparent circle was observed around the recombinant strains (Figure 3A), indicating their ability to grow with starch as the sole carbon source. The diameter of the transparent circle was 1.33 cm for the 1974-GA strain, 2.26 cm for the 1974-temA strain, and 2.68 ± 0.011 cm for the 1974-GA-temA strain. This result indicates that α-amylase has a stronger ability to hydrolyze starch than glucoamylase. The recombinant *S. cerevisiae* strain co-expressing α-amylase and glucoamylase exhibited the strongest ability to hydrolyze starch (Figure 3A).

The genetic stability of the strain 1974-GA-temA was evaluated by performing 30 serial passages on non-selective YPD plates. The average diameter of the transparent circle for the 10th, 20th, and 30th generations of the 1974-GA-temA strain was 2.68, 2.66, and 2.66 cm, respectively (Figure 3B). The consistent diameters observed after 30 generations indicate that the starch hydrolysis ability of the strain 1974-GA-temA has not changed. This result is due to the fact that the α-amylase and glucoamylase genes were integrated into specific sites on the *S. cerevisiae* chromosome, rather than expressed in plasmid form or integrated at the δ sites, both of which are prone to lose the copy number of foreign genes (Stepchenkova et al., 2023). Again we tested the *GA* and *temA* copy number in strain 1974-GA-temA after serial passages on non-selective YPD plates by using real-time PCR, eight copies of *GA* and eight copies of *temA* remained present in the strain (Supplementary Table 2). The results of the strain genetic stability testing indicate that the strain 1974-GA-temA has potential for industrial application.

**FIGURE 4:**
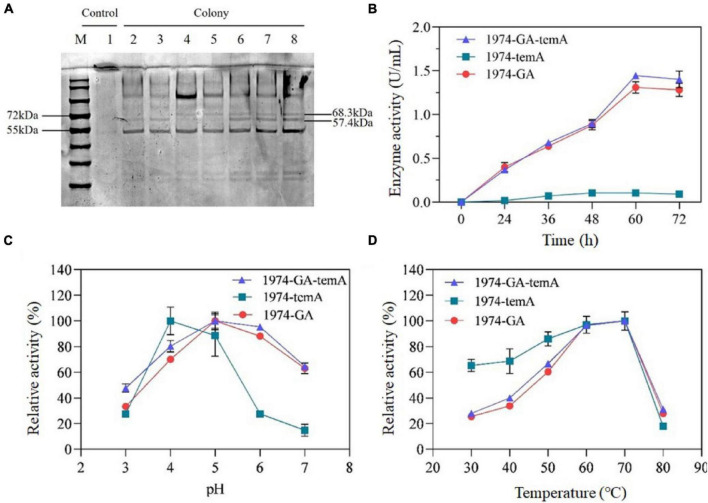
SDS-PAGE analysis of the expression of αα-amylase and glucoamylase **(A)** and enzymatic activity assays. Lane 1, 1974. Lane 2 to lane 3, 1974-GA. Lane 4 to lane 5, 1974-temA. Lane 6 to lane 8, 1974-GA-temA. Volumetric enzymatic activity profiles over time for the strain 1974-GA-temA **(B)**. Effect of pH **(C)** and temperature **(D)** on the enzymatic activity of recombinant enzymes. The original gel of Figure 4A was presented as Supplementary Figure 14. Data points represent the average of the results from three independent experiments.

α-Amylase and glucoamylase should be secreted into the medium to hydrolyze starch into glucose, which is then utilized by *S. cerevisiae*. SDS-PAGE analysis of the supernatant of fermentation broth was performed to identify the expression of α-amylase and glucoamylase directly. Based on the amino acid sequences, the predicted molecular weights of the unglycosylated GA and temA protein were 57.4 and 68.3 kDa, respectively. SDS-PAGE analysis of the supernatant indicated prominent bands for strains expressing GA, with the molecular size of the band being consistent with the predicted GA (Figure 4A). The strain 1974-temA exhibited clear protein bands between 55 and 72 kDa, whereas the strain 1974-GA-temA displayed the GA and temA protein bands (Figure 4A). The SDS-PAGE result indicated that α-amylase and glucoamylase were expressed and secreted into the medium.

The enzymatic activities of the recombinant strains are shown in Figure 4B. After 60 h of fermentation, the enzymatic activity stabilized. The α-amylase enzymatic activity reached 0.12 U/mL ± 0.01 U/mL, whereas the glucoamylase enzymatic activity reached 1.36 U/mL ± 0.07 U/mL. α-Amylase primarily hydrolyzes starch into dextrin and oligosaccharides, whereas glucoamylase primarily hydrolyzes dextrin and oligosaccharides into glucose (Cripwell et al., 2020). Consequently, the glucose released from starch by the enzyme of the strain 1974-GA is higher than that in the fermentation broth of the strain 1974-temA, resulting in lower α-amylase activity compared with glucoamylase activity. The strain 1974-GA-temA can simultaneously secrete α-amylase and glucoamylase. The fermentation broth of the strain 1974-GA-temA exhibits the highest glucose content, with enzymatic activity reaching 1.47 U/mL ± 0.10 U/mL (Figure 4B). The extracellular amylase activity of strain 1974-GA-temA was comparable with the strain ER T1 published in the previous report (Cripwell et al., 2019a).

As the combination of *T. emersonii*-derived α-amylase and *S. fibuligera-*derived glucoamylase has not been reported, the optimal pH and temperature for enzymatic activity were determined. A series of pH buffer systems (pH 3.0, pH 4.0, pH 5.0, pH 6.0, and pH 7.0) and temperature values (30°C, 40°C, 50°C, 60°C, 70°C, and 80°C) was tested (Figures 4C, D). The enzymatic activity of the recombinant enzyme was quantified by exhibiting the highest enzymatic activity designated as 100% relative enzymatic activity. The results indicated that the optimal pH for α-amylase was approximately 4.0, whereas that for glucoamylase was around 5.0 (Figure 4C). Furthermore, at a pH of 5.0, the optimal hydrolysis temperature for α-amylase and glucoamylase is approximately 60°C to 70°C, indicating that these enzymes exhibit relatively high hydrolysis temperatures (Figure 4D). α-Amylase and glucoamylase from different microbial sources have different optimum pH and temperature. For example, the optimal pH for *Aspergillus tubingensis* derived α-amylase and glucoamylase are 4.0, while the optimum temperatures are 60 and 70°C, respectively (Viktor et al., 2013). The combinations of α-amylase and glucoamylase from *T. emersonii* exhibited excellent raw starch hydrolyzed capability (Cripwell et al., 2019a), however, the optima pH and temperature for *T. emersonii* derived glucoamylase remains unclear.

### 3.3 Ethanol production from soluble starch with recombinant S. cerevisiae strains

**FIGURE 5:**
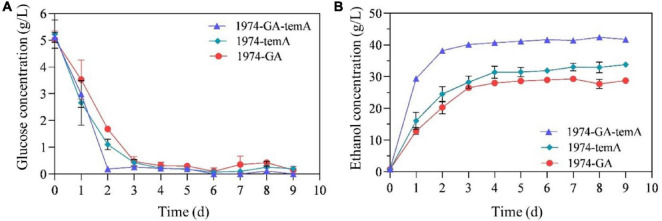
Fermentation of soluble starch by recombinant *S. cerevisiae* strain. **(A)** Residual glucose concentration during fermentation. **(B)** Ethanol concentration during fermentation. Data points represent the average of the results from three independent fermentation experiments.

The CBP simulation was initially conducted under fermentative conditions with recombinant *S. cerevisiae* strains utilizing 100 g/L soluble starch and 5 g/L glucose as the initial carbon source for cells. Samples were taken every 24 h to measure glucose and ethanol levels, and the iodine–starch reaction was used to determine the completion of starch hydrolysis. The strain 1974-GA-temA produced 42.2 g/L ± 0.52 g/L ethanol after 5 days of fermentation (Figure 5 and Table 3), corresponding to 70.59% of the theoretical yield. The strains 1974-GA and 1974-temA exhibited incomplete starch hydrolysis for 9 days and produced 28.4 g/L ± 0.57 g/L and 33.0 g/L ± 0.97 g/L ethanol, respectively (Figure 5). Although all three engineering strains can utilize soluble starch to produce ethanol, the strains 1974-GA and 1974-temA exhibited a weaker ability to convert starch. Therefore, complete hydrolysis of starch requires the synergistic action of α-amylase and glucoamylase.

**TABLE 3:** Fermentation of soluble starch or SPR to produce ethanol.

	1974-GA	1974-temA	1974-GA-temA	1974-GA-temA	1974-GA-temA	1974-GA-temA	1974-GA-temA	1974-GA-temA	1974-GA-temA	1974
Exogenous enzyme addition	0	0	0	0	20% Amy	20% GA	50% Amy	50% GA	50% Amy + 50% GA	100% Amy + 100% GA
Substrate concentration	100 g/L soluble starch + 5 g/L glucose	100 g/L soluble starch + 5 g/L glucose	100 g/L soluble starch + 5 g/L glucose	166.67 g/L SPR	166.67 g/L SPR	166.67 g/L SPR	166.67 g/L SPR	166.67 g/L SPR	166.67 g/L SPR	166.67 g/L SPR
Theoretical total glucose content (g/L)	116.11	116.11	116.11	106.31	106.31	106.31	106.31	106.31	106.31	106.31
Final ethanol (g/L)	28.37	33.03	42.22	32.15	34.22	50.55	34.60	51.32	51.15	50.68
Ethanol productivity (g/L h)	0.17	0.19	0.25	0.20	0.20	0.30	0.20	0.31	0.30	0.30
Estimated ethanol yield (g/g)	0.24	0.28	0.36	0.30	0.32	0.48	0.33	0.48	0.48	0.48
Estimated ethanol yield (% of theoretical yield)^a^	47.06	54.90	70.59	59.30	63.12	93.23	63.82	94.65	94.34	93.47

Amy, α-amylase; GA, Glucoamylase. ^a^The theoretical ethanol yield from glucose is 0.51. The data represent the average of three independent tests.

**FIGURE 6:**
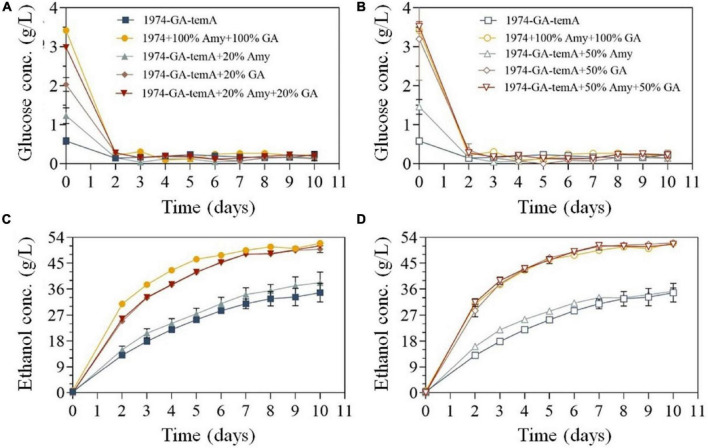
*S. cerevisiae* strains 1974 and 1974-GA-temA during fermentation in 250 mL fermentation flasks with SPR. The residual glucose concentrations **(A,B)** and ethanol concentrations **(C,D)** were monitored throughout fermentation. A total of 0.13 g of αα-amylase (3700 U/g) and 0.03 g of glucoamylase (100000 U/g) were used to supplement the CBP fermentation of strains, which were designated as 100% dosage. Data are the mean of three repeats showing standard deviation.

Previously, the enzymatic hydrolysates of SPR were used as a fermentation substrate to produce ethanol (Gou et al., 2023). Herein, SPR was directly used as the fermentation substrate. However, in the absence of α-amylase or glucoamylase, the strain 1974-GA-temA exhibited incomplete starch hydrolysis after 10 days (Figure 6), which could be attributed to the fermentation temperature not being optimal for the recombinant enzyme, resulting in a slow starch hydrolysis rate. Despite the advantages of CBP, recombinant proteins still need to be produced by the yeast strain before substrate hydrolysis can accelerate at the start of fermentation. Therefore, a portion of exogenous α-amylase or glucoamylase was supplemented. The results demonstrated that after adding exogenous α-amylase and glucoamylase to the initial strain 1974 for synchronous saccharification and fermentation for 8 days, the starch in the SPR was completely hydrolyzed (Figure 6 and Table 3). This reaction resulted in a final ethanol production of 50.68 g/L ± 0.07 g/L, with an ethanol yield of 0.48 g/g.

When the *S. cerevisiae* strain 1974-GA-temA was supplemented with exogenous α-amylase or glucoamylase, the ethanol production improved compared with that without exogenous α-amylase and/or glucoamylase amylase was added. Nevertheless, although exogenous α-amylase was added, the strain 1974-GA-temA cannot completely hydrolyze the starch after 10 days of fermentation. The results shown in Figure 6 demonstrate that the *S. cerevisiae* strain 1974-GA-temA can ferment starch after 10 days. This fermentation was achieved by adding either 20% exogenous glucoamylase or 20% exogenous α-amylase and 20% exogenous glucoamylase. In both cases, complete hydrolysis of starch was achieved. Furthermore, when 20% or 50% exogenous glucoamylase was supplemented, the starch was completely hydrolyzed on the 7th to 8th day of fermentation. In this case, the ethanol yield (50.55 g/L ± 0.13 g/L and 51.32 g/L ± 0.15 g/L, respectively) was comparable to the yields obtained when 100% α-amylase or 100% glucoamylase were added in the *S. cerevisiae* strain 1974 (Table 3). These findings indicate that exogenous glucoamylase exert a more pronounced influence on the fermentation of raw meal by the *S. cerevisiae* strain 1974-GA-temA. Considering its economic benefits, only 20% exogenous glucoamylase should be supplemented during the fermentation of SPR. Throughout the SPR fermentation cycle, the glucose in the culture medium was maintained at a very low level (Figure 6), indicating that the strain was metabolically active and could quickly utilize the decomposed glucose. In previous reports, as the fermentation time increased, the glucose content in the culture medium increased, indicating incomplete metabolism of the strain (Cripwell et al., 2019a,b). The results demonstrated that strain 1974-GA-temA constructed in this study showed great potential in SPR CBP bioethanol fermentation.

## 4 Conclusion

In this study, recombinant industrial ethanol-producing diploid *S. cerevisiae* strains with eight copies of α-amylase and eight copies of glucoamylase genes integration were successfully constructed. *In vitro* enzymatic activity analysis demonstrated that the optimal temperature for α-amylase and glucoamylase was 70°C, and the optimal pH for α-amylase and glucoamylase was 4.0 and 5.0, respectively. CBP fermentation of SPR with the recombinant amylolytic strain 1974-GA-temA demonstrated that the strain 1974-GA-temA produced 32.15 g/L ethanol in the absence of exogenous α-amylase and glucoamylase, corresponding to 59.30% of the theoretical yield. When 20% glucoamylase was added, ethanol production increased to 50.55 g/L, corresponding to 93.23% of the theoretical yield. This result is comparable to the parental strain 1974 fermented with 100% α-amylase and glucoamylase. Therefore, the yeast strain constructed in this study can be regarded as a potential CBP yeast for the commercial production of ethanol from SPR substrate.

## Data availability statement

The original contributions presented in this study are included in this article/Supplementary material, further inquiries can be directed to the corresponding authors.

## Author contributions

XW: Writing–review and editing, Writing–original draft, Supervision, Investigation, Funding acquisition, Conceptualization. NG: Writing–review and editing, Writing–original draft, Visualization, Methodology. JH: Writing–original draft, Methodology, Data curation. CG: Writing–original draft, Methodology. XX: Writing–original draft, Methodology. HZ: Methodology, Writing–review and editing. AL: Writing–review and editing, Funding acquisition. JH: Supervision, Funding acquisition, Writing–review and editing. MH: Writing–review and editing, Project administration. NL: Writing–review and editing, Writing–original draft, Project administration, Funding acquisition, Conceptualization.
