# Supplementary material for: Construction of an amylolytic Saccharomyces cerevisiae strain with high copies of α-amylase and glucoamylase genes integration for bioethanol production from sweet potato residue
Source: Front Microbiol. 2024 Aug 6;15:1419293. doi: 10.3389/fmicb.2024.1419293 (PMC11337298; doi:10.3389/fmicb.2024.1419293)
Supplement: Supplementary file 1 [file Data_Sheet_1.docx]

Supporting Information for

**Construction of an amylolytic *Saccharomyces cerevisiae* strain with high copies of α-amylase and glucoamylase genes integration for bioethanol production from sweet potato residue**

Xin Wang^1,2,3,4,5*^, Na Guo^2^, Jingting Hu^2^, Chenchen Gou^2^, Xinyue Xie^2^, Haobo Zheng^6^, Aimei Liao^2,5^, Jihong Huang^3,4,7^, Ming Hui^2,5^, Na Liu^2,5*^

^1^ National Engineering Research Center of Wheat and Corn Further Processing, Henan University of Technology, Zhengzhou 450001, Henan Province, China

^2^ College of Biological Engineering, Henan University of Technology, Zhengzhou 450001, Henan Province, China

^3^ School of Food and Pharmacy, Xuchang University, Xuchang 461000, Henan Province, China

^4^ Collaborative Innovation Center of Functional Food by Green Manufacturing, Xuchang 461000, Henan Province, China.

^5^ Henan Provincial Engineering Laboratory of Preservation and Breeding of Industrial Microbial Strains, Zhengzhou 450001, Henan Province, China

^6^ School of International Education, Henan University of Technology, Zhengzhou 450001, Henan Province, China

^7^ State Key Laboratory of Crop Stress Adaptation and Improvement, College of Agriculture, Henan University, Kaifeng 475004, Henan Province, China

*Corresponding authors:

Xin Wang (wangxin@haut.edu.cn )

Na Liu (liuna3456@163.com )

Mailing address: No. 100 Lianhua Street, Zhengzhou High-Tech Development Zone, Henan Province, P.R.China (450001)

Tel: 86-0371-67756843

**Medium formulation**

LB: Yeast extract 5 g/L, Tryptone 10 g/L, NaCl 5 g/L. Solid agar plates were prepared by adding 1.5% agar.

YPD: Yeast extract 10 g/L, Tryptone 20 g/L, D-glucose 20 g/L. Solid agar plates were prepared by adding 1.5% agar.

YPD-G418: Yeast extract 10 g/L, Tryptone 20 g/L, D-glucose 20 g/L, G418 300 μg/mL. Solid agar plates were prepared by adding 1.5% agar.

YPD-HYG: Yeast extract 10 g/L, Tryptone 20 g/L, D-glucose 20 g/L, hygromycin 400 μg/mL. Solid agar plates were prepared by adding 1.5% agar.

YPD-G418-HYG: Yeast extract 10 g/L, Tryptone 20 g/L, D-glucose 20 g/L, G418 300 μg/mL, hygromycin 400 μg/mL. Solid agar plates were prepared by adding 1.5% agar.

**Plasmids construction**

**X-2 site GA integration plasmid pX-2-2GA construction**

For the X-2 site specific two copies of GA containing plasmid pX-2-2GA construction, the backbone fragment including the yeast CEN/ARS sequence, *E. coli* pUC replication origin, the ampicillin selection marker gene, and the URA3 selection marker gene, flanked with *Not*I sites were amplified from plasmid pHCas9 (Wang, Yang et al. 2019) using primer pairs h-bb-X-2D-F1 and h-bb-X-2U-R1.

The X-2 site specific 200 bp length up homology arm flanked with *Not*I and *Sgs*I sites were amplified from 1974 genomic DNA with primers hX-2U-bb-F1 and hX-2U-X-2d-R1, the 200 bp length down homology arm flanked with *Not*I and *Mss*I sites were also amplified from the same template using primers hX-2D-X-2U-F1/hX-2D-bb-R1. The up and down homology arms were fused with primers hX-2U-bb-F1 and hX-2D-bb-R1, and the resulting 400 bp length homology arms were ligated with the backbone fragment to make plasmid pX-2-SgsI-MssI.

To add the *Sac*I and *Xho*I restriction sites between the *Sgs*I and *Mss*I sites, the whole plasmid pX-2-SgsI-MssI was amplified using primer pairs X-2-SacI-XhoI-F1 and X-2-SacI-MssI-R1, the resulting fragment was digested with *Xho*I or *Sac*I and ligated to make plasmid pX-2-*Sac*I-*Xho*I.

Using the 1974 genome as template, the GAP promoter and CYC1 terminator were amplified using the GAPp-R1/GA-GAPp-F1 pairs and X-2-CYC1t-F1/GA-CYC1t-R1, respectively. GA was amplified from the previous constructed plasmid pYIE2-2GA-δ using GAPp-GA-R1/CYC1t-GA-F1 primer pairs. The GAP promoter, GA gene, CYC1 terminator were fused by the overlap PCR flanked with the *Xho*I and *Sac*I restriction sites. The resulting GA expression cassette was ligated to pX-2-SacI-XhoI which was cutted with XhoI and SacI to make plasmid pX-2-GA.

Another copy of GA was also constructed, the TEF1 promoter and ADH1 terminator were amplified using the X-2-TEF1p-F1/GA-TEF1p-R1 pairs and GA-ADH1t-F1/X-2-ADH1t-R1, respectively. GA was amplified from the previous constructed plasmid pYIE2-2GA-δ using ADH1t-GA-R1/TEF1p-GA-F1 primer pairs. The TEF1 promoter, GA gene, ADH1 terminator were fused by the overlap PCR flanked with the *Sac*I and *Mss*I restriction sites. The resulting GA expression cassette was ligated to pX-2-GA which was cutted with *Sac*I and *Mss*I to make the final plasmid pX-2-2GA.

**XII-5 site GA integration plasmid pXII-5-2GA construction**

Similar with the construction of plasmid pX-2-2GA, for the XII-5 site specific two copies of GA containing plasmid pXII-5-2GA construction, the backbone fragment including the yeast CEN/ARS sequence, *E. coli* pUC replication origin, the ampicillin selection marker gene, and the URA3 selection marker gene, flanked with *Not*I sites were amplified from plasmid pHCas9 using primer pairs h-bb-XII-5D-F1/h-bb-XII-5U-R1.

The XII-5 site specific 200 bp length up homology arm flanked with *Not*I and *Sgs*I sites were amplified from 1974 genomic DNA with primers h-XII-5U-bb-F1/h-XII-5U-XII-5d-R1, the 200 bp length down homology arm flanked with *Not*I and *Mss*I sites were also amplified from the same template using primers h-XII-5D-XII-5U-F1/h-XII-5D-bb-R1. The up and down homology arms were fused with primers h-XII-5U-bb-F1 and h-XII-5D-bb-R1, and the resulting 400 bp length homology arms were ligated with the backbone fragment to make plasmid pXII-5-SgsI-MssI.

To add the *Bcu*I and *Xho*I restriction sites between the *Sgs*I and *Mss*I sites, the whole plasmid pXII-5-SgsI-MssI was amplified using primer pairs XII-5-XhoI-SgsI-F1 and XII-5-MssI-BcuI-R1, the resulting fragment was digested with *Xho*I or *Bcu*I and ligated to make plasmid pXII-5-BcuI-XhoI.

Using the 1974 genome as template, the GAP promoter and CYC1 terminator were amplified using the GAPp-R1/GA-GAPp-F1 pairs and XII-5-CYC1t-F1/GA-CYC1t-R1, respectively. GA was amplified from the previous constructed plasmid pYIE2-2GA-δ using GAPp-GA-R1/CYC1t-GA-F1 primer pairs. The GAP promoter, GA gene, CYC1 terminator were fused by the overlap PCR flanked with the *Xho*I and *Bcu*I restriction sites. The resulting GA expression cassette was ligated to pXII-5-BcuI-XhoI which was cutted with *Xho*I and *Bcu*I to make plasmid pXII-5-GA.

Another copy of GA was also constructed, the TEF1 promoter and ADH1 terminator were amplified using the XII-5-TEF1p-F1/GA-TEF1p-R1 pairs and GA-ADH1t-F1/XII-5-ADH1t-R1, respectively. GA was amplified from the previous constructed plasmid pYIE2-2GA-δ using ADH1t-GA-R1/TEF1p-GA-F1 primer pairs. The TEF1 promoter, GA gene, ADH1 terminator were fused by the overlap PCR flanked with the *Bcu*I and *Mss*I restriction sites. The resulting GA expression cassette was ligated to pXII-5-GA which was cutted with *Bcu*I and *Mss*I to make the final two copies of GA containing plasmid pXII-5-2GA.

**X-3 site temA integration plasmid pX-3-2temA construction**

For the X-3 site specific two copies of temA containing plasmid pX-3-2GA construction, the backbone fragment including the yeast CEN/ARS sequence, *E. coli* pUC replication origin, the ampicillin selection marker gene, and the URA3 selection marker gene, flanked with *Not*I sites were amplified from plasmid pHCas9 using primer pairs h-bb-X-3D-F1/h-bb-X-3U-R1.

The X-3 site specific 200 bp length up homology arm flanked with *Not*I and *Sgs*I sites were amplified from 1974 genomic DNA with primers h-X-3U-bb-F1/h-X-3U-X-3D-R1, the 200 bp length down homology arm flanked with *Not*I and *Mss*I sites were also amplified from the same template using primers h-X-3D-X-3U-F1/h-X-3D-bb-R1. The up and down homology arms were fused with primers h-X-3U-bb-F1/h-X-3D-bb-R1, and the resulting 400 bp length homology arms were ligated with the backbone fragment to make plasmid pX-3-SgsI-MssI.

To add the *Bcu*I and *Xho*I restriction sites between the *Sgs*I and *Mss*I sites, the whole plasmid pX-3-SgsI-MssI was amplified using primer pairs X-3-BcuI-MssI-F1/ X-3-XhoI-SgsI-R1, the resulting fragment was digested with *Bcu*I or *Xho*I and ligated to make plasmid pX-3-BcuI-XhoI.

Using the 1974 genome as template, the GAP promoter and CYC1 terminator were amplified using the XI-2-GAPp-F1/D-temA-GAPp-R1 pairs and D-temA-CYC1t-F1/XII-5-CYC1t-R1, respectively. temA was amplified from the plasmid ptemA using D-GAPp-temA-F1/D-CYC1t-temA-R1 primer pairs. The GAP promoter, temA gene, CYC1 terminator were fused by the overlap PCR flanked with the *Bcu*I and *Sgs*I restriction sites. The resulting temA expression cassette was ligated to pX-3-BcuI-XhoI which was cutted with *Bcu*I and *Sgs*I to make plasmid pX-3-temA.

Another copy of temA was also constructed, the TEF1 promoter and ADH1 terminator were amplified using the D-temA-TEF1p-F1/X-3-MssI-R1 pairs and XII-5-BcuI-R1/D-temA-ADH1t-R1, respectively. temA was amplified from the plasmid ptemA using D-ADH1t-temA-F1/D-TEF1p-temA-R1 primer pairs. The TEF1 promoter, GA gene, ADH1 terminator were fused by the overlap PCR flanked with the *Bcu*I and *Mss*I restriction sites. The resulting temA expression cassette was ligated to pX-3-temA which was cutted with *Bcu*I and *Mss*I to make the final two copies of GA containing plasmid pX-3-2temA.

**XI-2 site temA integration plasmid pXI-2-2temA construction**

For the XI-2 site specific two copies of temA containing plasmid pXI-2-2temA construction, the backbone fragment including the yeast CEN/ARS sequence, *E. coli* pUC replication origin, the ampicillin selection marker gene, and the URA3 selection marker gene, flanked with *Not*I sites were amplified from plasmid pHCas9 using primer pairs h-bb-XI-2D-F1/h-bb-XI-2U-R1.

The XI-2 site specific 200 bp length up homology arm flanked with *Not*I and *Sgs*I sites were amplified from 1974 genomic DNA with primers h-XI-2U-bb-F1/h-XI-2U-XI-2d-R1, the 200 bp length down homology arm flanked with *Not*I and *Mss*I sites were also amplified from the same template using primers h-XI-2D-XI-2U-F1/h-XI-2D-bb-R1. The up and down homology arms were fused with primers h-XI-2U-bb-F1/h-XI-2D-bb-R1, and the resulting 400 bp length homology arms were ligated with the backbone fragment to make plasmid pXI-2-SgsI-MssI.

To add the *Sac*I and *Xho*I restriction sites between the *Sgs*I and *Mss*I sites, the whole plasmid pXI-2-SgsI-MssI was amplified using primer pairs XI-2-SacI-MssI-F1/ XI-2-XhoI-SgsI-R1, the resulting fragment was digested with *Bcu*I and ligated to make plasmid pXI-2-SacI-XhoI.

Using the 1974 genome as template, the GAP promoter and CYC1 terminator were amplified using the XI-2-GAPp-F1/D-temA-GAPp-R1 pairs and D-temA-CYC1t-F1/X-2-CYC1t-F1, respectively. temA was amplified from the plasmid ptemA using D-GAPp-temA-F1/D-CYC1t-temA-R1 primer pairs. The GAP promoter, temA gene, CYC1 terminator were fused by the overlap PCR flanked with the *Sac*I and *Sgs*I restriction sites. The resulting temA expression cassette was ligated to pXI-2-BcuI-XhoI which was cutted with *Sac*I and *Sgs*I to make plasmid pXI-2-temA.

Another copy of temA was also constructed, the TEF1 promoter and ADH1 terminator were amplified using the D-temA-TEF1p-F1/XI-2-MssI-R1 pairs and X-2-SacI-R1/D-temA-ADH1t-R1, respectively. temA was amplified from the plasmid ptemA using D-ADH1T-temA-F1/D-TEF1p-temA-R1 primer pairs. The TEF1 promoter, GA gene, ADH1 terminator were fused by the overlap PCR flanked with the *Bcu*I and *Mss*I restriction sites. The resulting temA expression cassette was ligated to pXI-2-temA which was cutted with *Sac*I and *Mss*I to make the final two copies of GA containing plasmid pXI-2-2temA.

**Strains construction**

The yeast transformation was performed using standard procedure described previously (Gietz and Schiestl 2007). The Cas9 expression plasmid pHCas9 was firstly introduced into the strain 1974 to make strain 1974/Cas9, GA gene containing plasmids pXII-5-2GA and pX-2-2GA were both digested with *Not*I, the two copies of GA expression cassettes (4605 bp) were isolated from agarose gel and purified. Next, the 2-3 μg of *Not*I-linearized pXII-5-2GA plasmid, 2-3 μg of *Not*I-linearized pX-2-2GA plasmid, and 2 μg of gRNA expression plasmid pYES2-XII-5-X-2gRNA-hyg (Wang, Yang et al. 2019) were co-transformed into the pHCas9 plasmid harboring strains 1974/Cas9. The successful eight copies of GA expression cassettes integration colonies were confirmed by diagnostic PCR and named 1974-GA.

Next the pYES2-XII-5-X-2gRNA-hyg plasmid was removed by serial transfer in non-selective YPD plate for 10-15 times to prepare for the next integration. The 2-3 μg of *Not*I-linearized pXI-2-2temA plasmid, 2-3 μg of *Not*I-linearized pX-3-2temA plasmid, and 2 μg of gRNA expression plasmid pYES2-X-3-XI-2gRNA-hyg (Wang, Yang et al. 2019) were co-transformed into the strain 1974-GA. The successful eight copies of temA expression cassettes integration colonies were confirmed by diagnostic PCR and named 1974-GA-temA.

For the construction of temA gene integration strain 1974-temA, The 2-3 μg of *Not*I-linearized pXI-2-2temA plasmid, 2-3 μg of *Not*I-linearized pX-3-2temA plasmid, and 2 μg of gRNA expression plasmid pYES2-X-3-XI-2gRNA-hyg were co-transformed into the strain 1974/Cas9. The successful eight copies of temA expression cassettes integration colonies were confirmed by diagnostic PCR and named 1974-temA.


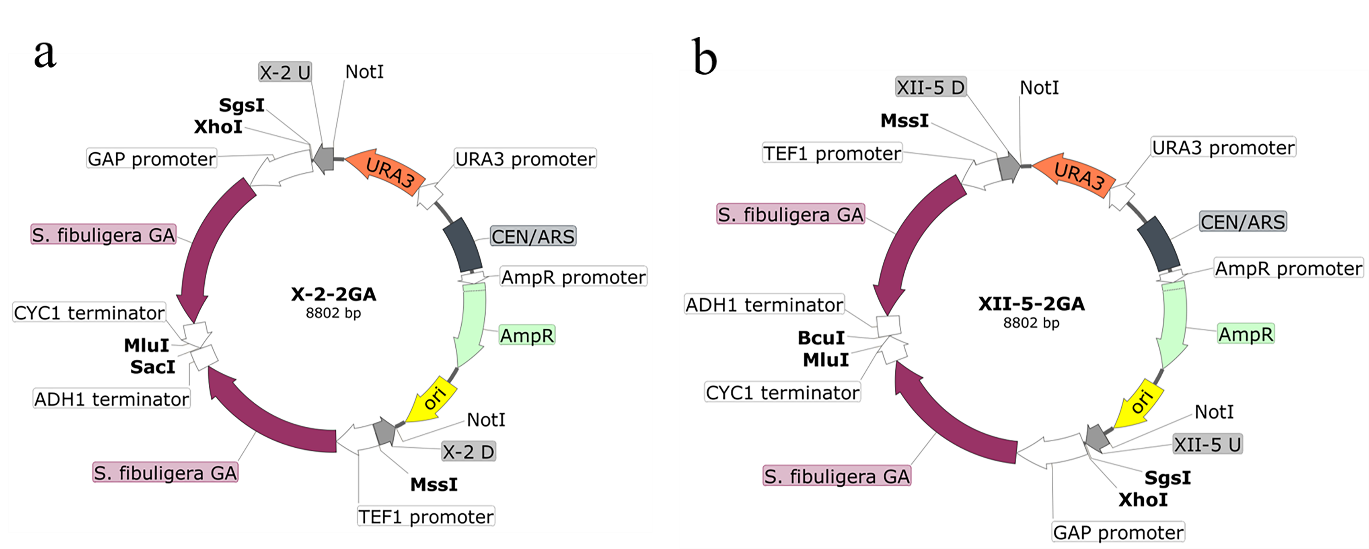


**Fig. S1** The map of the plasmid pX-2-2GA (a) and pXII-5-2GA (b).


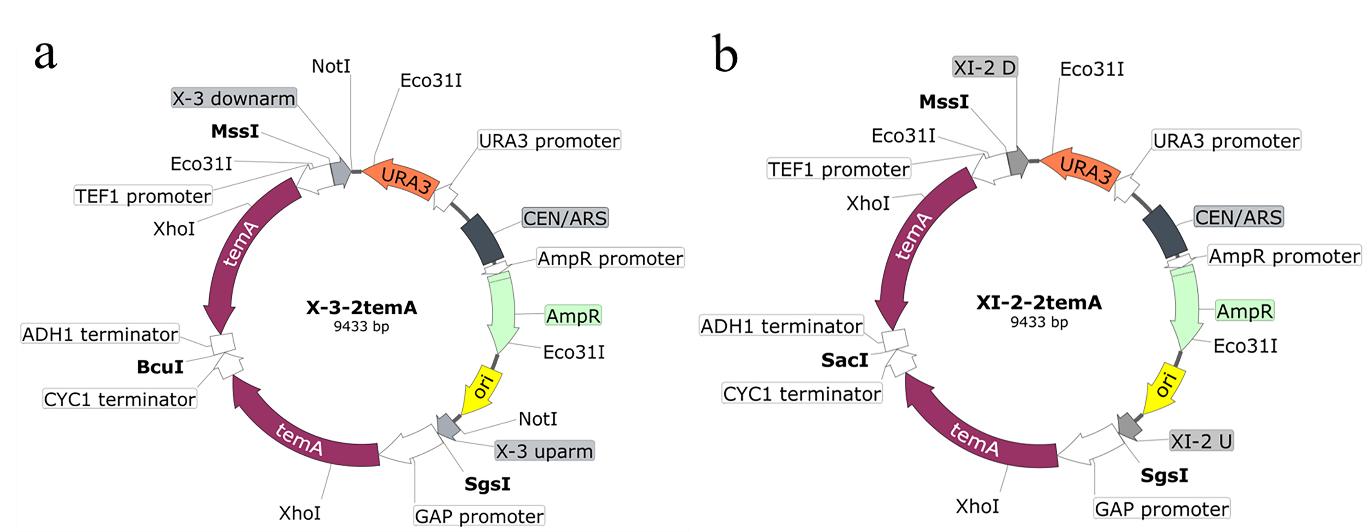


**Fig. S2** The map of the plasmid pX-3-2temA (a) and pXI-2-2temA (b).

.

**
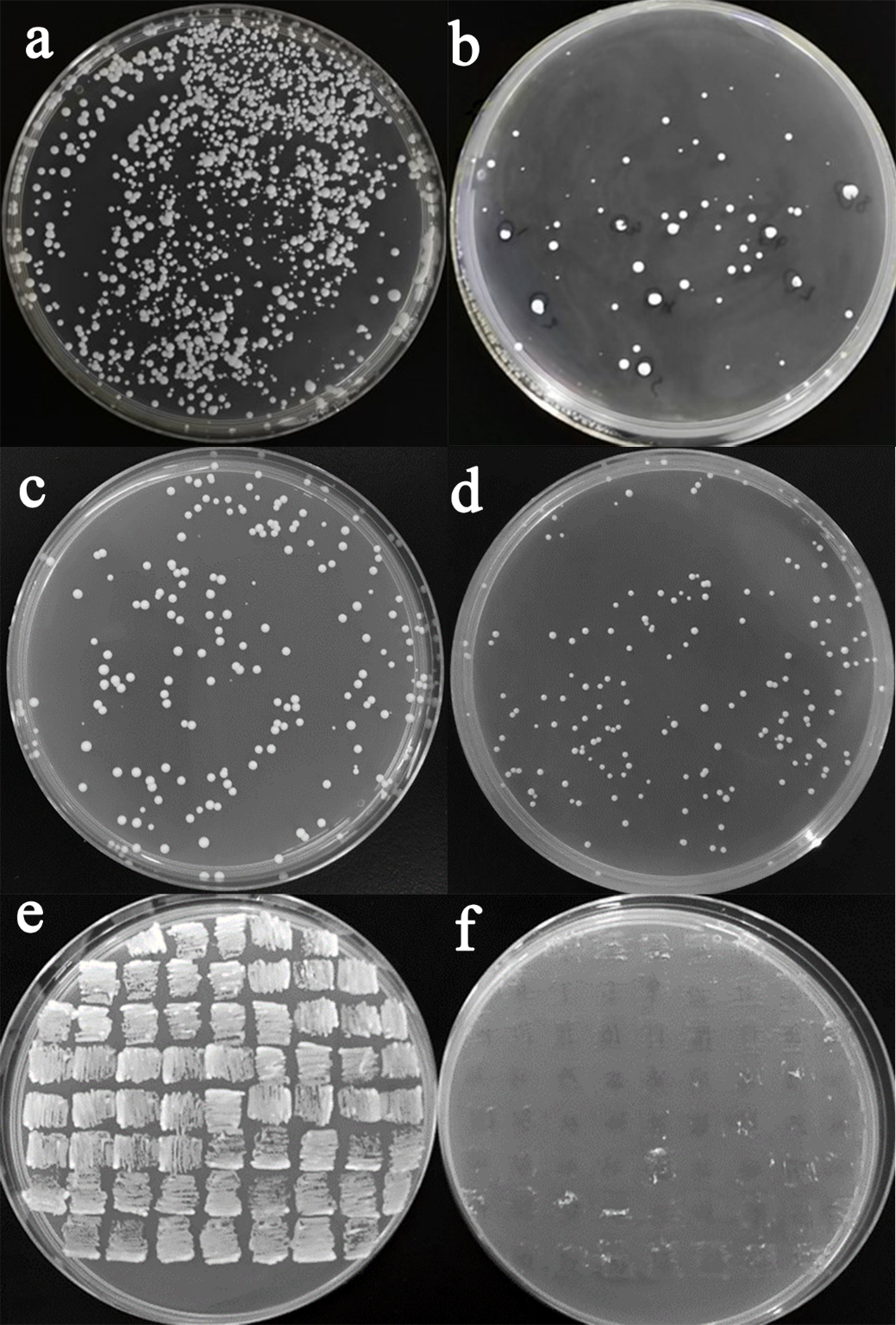
**

**Fig. S3** Transformants on the selective agar plates. a: 1974/cas9 strains. b: 1974-GA strains. c: 1974-temA strains. d: 1974-GA-temA strains. e (YPD-G418 plate) and f (YPD-HYG plate): validation of pYES2-XII-5-X-2gRNA-hyg plasmid removed through serial transfer in non-selective YPD plate.

**
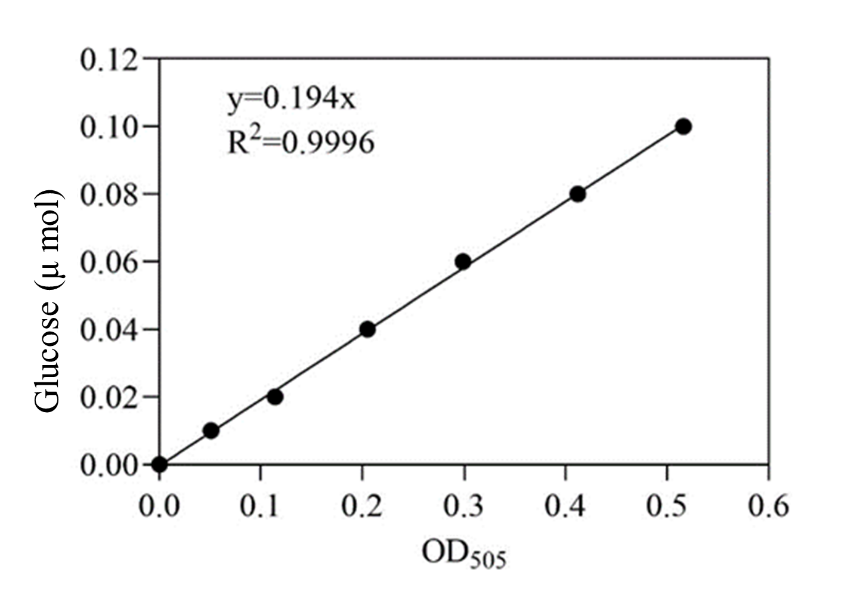
**

**Fig. S4** A standard curve of the glucose. Reducing sugar concentration was determined from the standard curve of glucose and multiplied by dilution factor.

**Table S1 Primers used in this study.**

| **Primer name** | **sequence** | **Purpose** |
| --- | --- | --- |
| h-bb-X-2D-F1 | tgtgcggccgcgtgcggtatttcacaccgc | Amplification of yeast CEN/ARS sequence, pUC replication origin, ampicillin selection marker gene, and the URA3 selection marker gene, NotI site was added. |
| h-bb-X-2U-R1 | atcgcggccgctacggttatccacagaatcag | Amplification of yeast CEN/ARS sequence, pUC replication origin, ampicillin selection marker gene, and the URA3 selection marker gene, NotI site was added. |
| h-X-2U-bb-F1 | gtagcggccgcgatcttctatcctctttagg | Amplification of X-2 up homology arm with *Not*I and *Sgs*I restriction sites added. |
| h-X-2U-X-2D-R1 | gtttaaacatatatggcgcgccgccgattatgcaggcctagaccc | Amplification of X-2 up homology arm with *Not*I and *Sgs*I restriction sites added. |
| h-X-2D-X-2U-F1 | ggcgcgccatatatgtttaaacccatctatgctgaagatttatc | Amplification of X-2 down homology arm with *Not*I and *Mss*I restriction sites added. |
| h-X-2D-bb-R1 | cacgcggccgcacactattcaggggaaggctac | Amplification of X-2 down homology arm with *Not*I and *Mss*I restriction sites added. |
| X-2-*Sac*1-*Xho*1-F1 | gagctcataatctcgagtatatggcgcgccgccgattatgc | Amplification of the whole plasmid pX-2-*Sgs*I-*Mss*I with *Xho*I and *Sac*I restriction sites added. |
| X-2-*Sac*1-*Mss*1-R1 | tatactcgagattatgagctctatatgtttaaacccatctatgctg | Amplification of the whole plasmid pX-2-*Sgs*I-*Mss*I with *Xho*I and *Sac*I restriction sites added. |
| GAPp-R1 | atatactcgagtcattatcaatactgccatttcaaag | Amplification of the GAP promoter. |
| GA-GAPp-F1 | caatctgatcattttgtttgtttatgtgtgtttattc | Amplification of the GAP promoter. |
| X-2-CYC1t-F1 | atatagagctcgcaaattaaagccttcgagcgtcccaaaac | Amplification of the CYC1 terminator. |
| GA-CYC1t-R1 | gttgatcgaattattgtaatcatgtaattagttatgtcac | Amplification of the CYC1 terminator. |
| GAPp-GA-R1 | cataaacaaacaaaatgatcagattgaccgttttc | Amplification of the GA gene. |
| CYC1t-GA-F1 | cataactaattacatgattacaataattcgatcaacttg | Amplification of the GA gene. |
| X-2-TEF1p-F1 | gatgggtttaaaccatagcttcaaaatgtttctactc | Amplification of the TEF1 promoter. |
| GA-TEF1p-R1 | cggtcaatctgatcatcttagattagattgctatgc | Amplification of the TEF1 promoter. |
| GA-ADH1t-F1 | tgatcgaattattgtaagcgaatttcttatgatttatg | Amplification of the ADH1 terminator. |
| X-2-ADH1t-R1 | tttgcgagctcccggtagaggtgtggtcaataag | Amplification of the ADH1 terminator. |
| ADH1t-GA-R1 | aatcataagaaattcgcttacaataattcgatcaacttg | Amplification of the GA gene. |
| TEF1p-GA-F1 | gcaatctaatctaagatgatcagattgaccgttttc | Amplification of the GA gene. |
| h-bb-XII-5D-F1 | gtagcggccgcgtgcggtatttcacaccgc | Amplification of yeast CEN/ARS sequence, pUC replication origin, ampicillin selection marker gene, and the URA3 selection marker gene, *Not*I site was added. |
| h-bb-XII-5U-R1 | ctcgcggccgctacggttatccacagaatcag | Amplification of yeast CEN/ARS sequence, pUC replication origin, ampicillin selection marker gene, and the URA3 selection marker gene, *Not*I site was added. |
| h-XII-5U-bb-F1 | gtagcggccgcgagtcactgacagccaccgc | Amplification of XII-5 up homology arm with *Not*I and *Mss*I restriction sites added. |
| h-XII-5U-XII-5d-R1 | gtttaaacatatatggcgcgcccagttcgctgtcactgaactaaaac | Amplification of XII-5 up homology arm with *Not*I and *Mss*I restriction sites added. |
| h-XII-5D-XII-5U-F1 | ggcgcgccatatatgtttaaacacggtagagcggaagaacag | Amplification of XII-5 down homology arm with *Not*I and *Mss*I restriction sites added. |
| h-XII-5D-bb-R1 | cacgcggccgctacatcaggacagtagtacc | Amplification of XII-5 down homology arm with *Not*I and *Mss*I restriction sites added. |
| XII-5-*Mss*1-*Bcu*1-R1 | tactcgagattatactagttatatgtttaaacacggtagagcgg | Amplification of the whole plasmid pXII-5-*Sgs*I-*Mss*I with *Xho*I and *Bcu*I restriction sites added. |
| XII-5-*Xho*1-*Sgs*1-F1 | actagtataatctcgagtatatggcgcgcccagttcgctgtcac | Amplification of the whole plasmid pXII-5-*Sgs*I-*Mss*I with *Xho*I and *Bcu*I restriction sites added. |
| XII-5-CYC1t-F1 | atataactagtgcaaattaaagccttcgagcgtccc | Amplification of the CYC1 terminator. |
| XII-5-TEF1p-F1 | accgtgtttaaaccatagcttcaaaatgtttctactcc | Amplification of the TEF1 promoter. |
| GA-TEF1p-R1 | cggtcaatctgatcatcttagattagattgctatgc | Amplification of the TEF1 promoter. |
| GA-ADH1t-F1 | tgatcgaattattgtaagcgaatttcttatgatttatg | Amplification of the ADH1 terminator. |
| XII-5-ADH1t-R1 | tttgcactagtccggtagaggtgtggtcaataag | Amplification of the ADH1 terminator. |
| h-bb-X-3D-F1 | aaagcggccgcgtgcggtatttcacaccgc | Amplification of yeast CEN/ARS sequence, pUC replication origin, ampicillin selection marker gene, and the URA3 selection marker gene, *Not*I site was added. |
| h-bb-X-3U-R1 | tatgcggccgctacggttatccacagaatcag | Amplification of yeast CEN/ARS sequence, pUC replication origin, ampicillin selection marker gene, and the URA3 selection marker gene, *Not*I site was added. |
| h-X-3U-bb-F1 | gtagcggccgcatatggcatcgttaaaac | Amplification of X-3 up homology arm with *Not*I and *Sgs*I restriction sites added. |
| h-X-3U-X-3D-R1 | gtttaaacatatatggcgcgcctctcgtatgtcggctctcg | Amplification of X-3 up homology arm with *Not*I and *Sgs*I restriction sites added. |
| h-X-3D-X-3U-F1 | ggcgcgccatatatgtttaaacccacttttcaatgaaacgg | Amplification of X-3 down homology arm with *Not*I and *Mss*I restriction sites added. |
| h-X-3D-bb-R1 | cacgcggccgcttttggttgattatccggcttc | Amplification of X-3 down homology arm with *Not*I and *Mss*I restriction sites added. |
| X-3-*Bcu*1-*Mss*1-F1 | atatactcgagataatactagttatatgtttaaacccacttttcaatg | Amplification of the whole plasmid pX-3-*Sgs*I-*Mss*I with *Xho*I and *Bcu*I restriction sites added. |
| X-3-*Xho*1-*Sgs*1-R1 | atataactagtattatctcgagtatatggcgcgcctctcgtatgtcggc | Amplification of the whole plasmid pX-3-*Sgs*I-*Mss*I with *Xho*I and *Bcu*I restriction sites added. |
| XI-2-GAPp-F1 | tataggcgcgcctcattatcaatactgccatttc | Amplification of the GAP promoter. |
| D-temA-GAPp-R1 | aaaggcgtcattttgtttgtttatgtgtg | Amplification of the GAP promoter. |
| D-temA-CYC1t-F1 | acacatggagatagtcatgtaattagttatgtc | Amplification of the CYC1 terminator. |
| XII-5-CYC1t-R1 | atataactagtgcaaattaaagccttcgagcgtccc | Amplification of the CYC1 terminator. |
| D-GAPp-temA-F1 | caaacaaaatgacgcctttcgtcctc | Amplification of the temA gene. |
| D-CYC1t-temA-R1 | taattacatgactatctccatgtgtcgac | Amplification of the temA gene. |
| D-temA-TEF1p-F1 | aaggcgtcatcttagattagattgctatg | Amplification of the TEF1 promoter. |
| X-3-*Mss*1-R1 | agtgggtttaaaccatagcttcaaaatgtttc | Amplification of the TEF1 promoter. |
| XII-5-*Bcu*1-R1 | atttgcactagtccggtagaggtgtggtcaataagag | Amplification of the ADH1 terminator. |
| D-temA-ADH1t-R1 | acacatggagataggcgaatttcttatgatttatg | Amplification of the ADH1 terminator. |
| D-ADH1t-temA-F1 | aaattcgcctatctccatgtgtcgac | Amplification of the temA gene. |
| D-TEF1p-temA-R1 | taatctaagatgacgcctttcgtcctc | Amplification of the temA gene. |
| h-bb-XI-2D-F1 | tttgcggccgcgtgcggtatttcacaccgc | Amplification of yeast CEN/ARS sequence, pUC replication origin, ampicillin selection marker gene, and the URA3 selection marker gene, *Not*I site was added. |
| h-bb-XI-2U-R1 | aaagcggccgctacggttatccacagaatcag | Amplification of yeast CEN/ARS sequence, pUC replication origin, ampicillin selection marker gene, and the URA3 selection marker gene, *Not*I site was added. |
| h-XI-2U-bb-F1 | gtagcggccgctttttgttcggtaatagcacg | Amplification of XI-2 up homology arm with *Not*I and *Sgs*I restriction sites added. |
| h-XI-2U-XI-2D-R1 | gtttaaacatatatggcgcgcccgagctttgcttgtggaagttcatg | Amplification of XI-2 up homology arm with *Not*I and *Sgs*I restriction sites added. |
| h-XI-2D-XI-2U-F1 | ggcgcgccatatatgtttaaacgggtacacacgactagtgctttc | Amplification of XI-2 down homology arm with *Not*I and *Mss*I restriction sites added. |
| h-XI-2D-bb-R1 | cacgcggccgcaaactttaatcaagaaatccg | Amplification of XI-2 down homology arm with *Not*I and *Mss*I restriction sites added. |
| XI-2-*Sac*1-*Mss*1-F1 | atatactcgagataatgagctctatatgtttaaacgggtacacacgac | Amplification of the whole plasmid pXI-2-*Sac*I-*Xho*I with *Sac*I and *Xho*I restriction sites added. |
| XI-2-*Xho*1-*Sgs*1-R1 | atatagagctcattatctcgagtatatggcgcgcccgagctttgc | Amplification of the whole plasmid pXI-2-*Sac*I-*Xho*I with *Sac*I and *Xho*I restriction sites added. |
| XI-2-*Mss*1-R1 | tacccgtttaaaccatagcttcaaaatgtttctactc | Amplification of the TEF1 promoter. |
| QSAct1-F | ggcttctttgactaccttcca | Forward real-time quantitative PCR primer for *ACT1* gene |
| QSAct1-R | agaaacacttgtggtgaacga | Reverse real-time quantitative PCR primer for *ACT1* gene |
| qtemA-f1 | TCCGTCTTACACTTGTCCCTACC | Forward real-time quantitative PCR primer for *temA* gene |
| qtemA-r1 | CTGCCGCTAGAGGATTCAAACG | Reverse real-time quantitative PCR primer for *temA* gene |
| qGA-f1 | TCATTCTTTCTCCAACACTACC | Forward real-time quantitative PCR primer for *GA* gene |
| qGA-r1 | GAATCGAAGTTACCGGATGG | Reverse real-time quantitative PCR primer for *GA* gene |

Table S2 GA and temA genes copy number in strain 1974-GA-temA after 30 serial passages on non-selective YPD plates

| Genes | 1974-GA-temA |
| --- | --- |
| *GA* | 8.04 ± 0.17 |
| *temA* | 8.30 ± 0.15 |

*Note: The values are the averages calculated from C_t_ values measured in triplicate reactions with recombinant strains’ DNA.*

**References**

Wang, X., J. J. Yang, S. Yang and Y. Jiang (2019). "Unraveling the genetic basis of fast l-arabinose consumption on top of recombinant xylose-fermenting *Saccharomyces cerevisiae*." Biotechnology and Bioengineering **116**(2): 283-293.
