# Supplementary figures and images for: Construction of an amylolytic Saccharomyces cerevisiae strain with high copies of α-amylase and glucoamylase genes integration for bioethanol production from sweet potato residue
Source: Front Microbiol. 2024 Aug 6;15:1419293. doi: 10.3389/fmicb.2024.1419293 (PMC11337298; doi:10.3389/fmicb.2024.1419293)

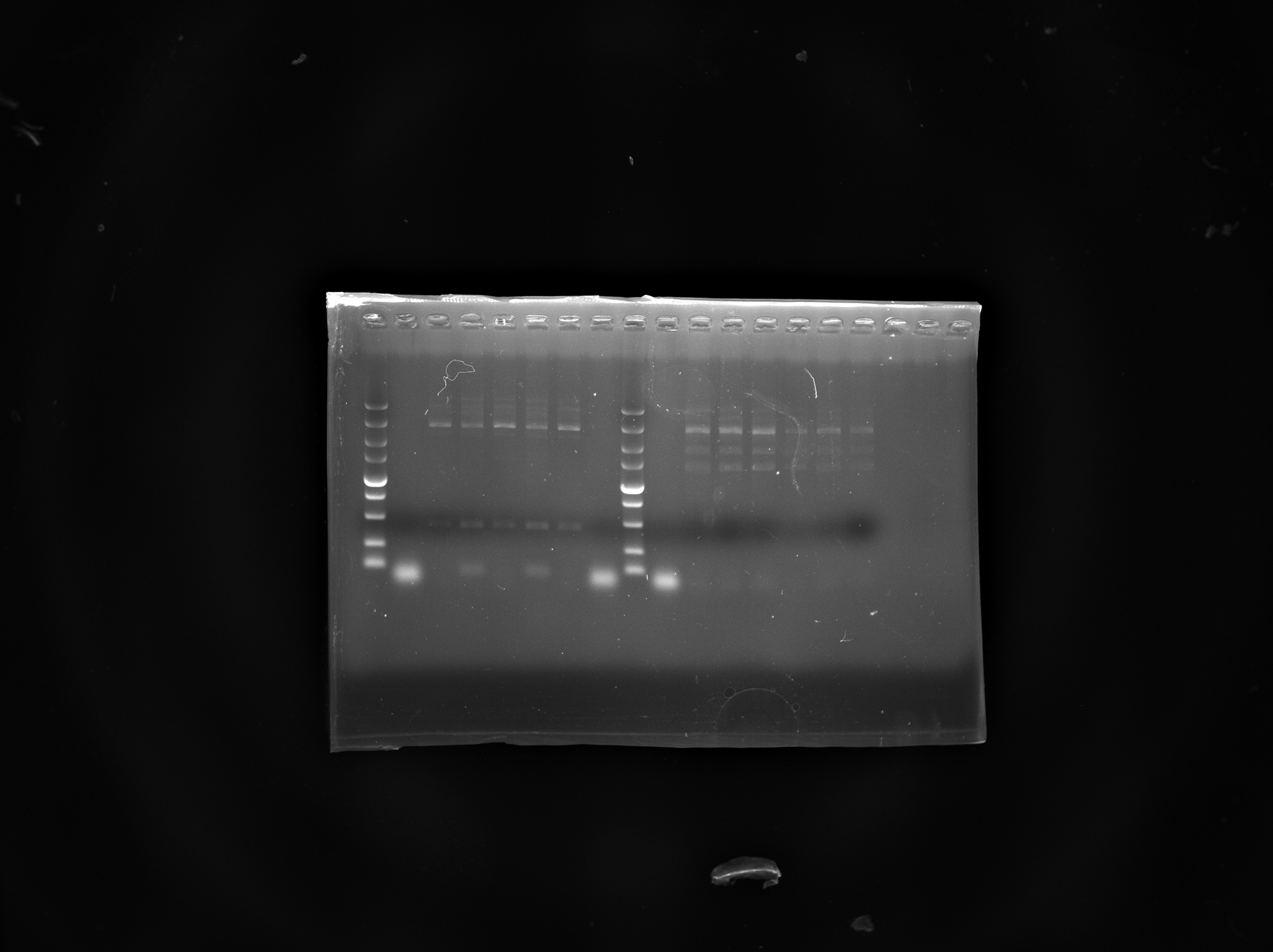

Supplement: Supplementary file 2 [file Image_1.TIF]

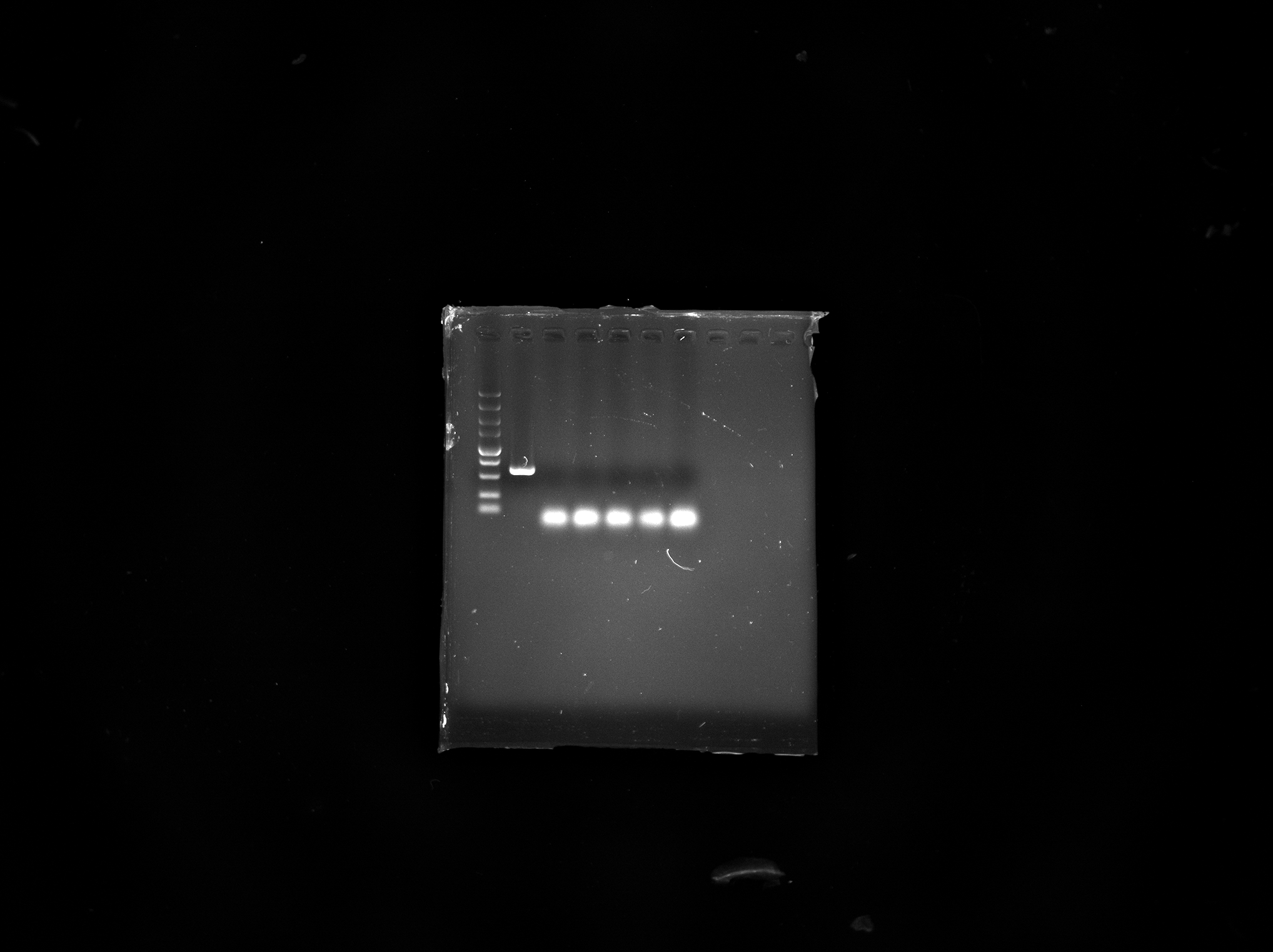

Supplement: Supplementary file 3 [file Image_2.TIF]

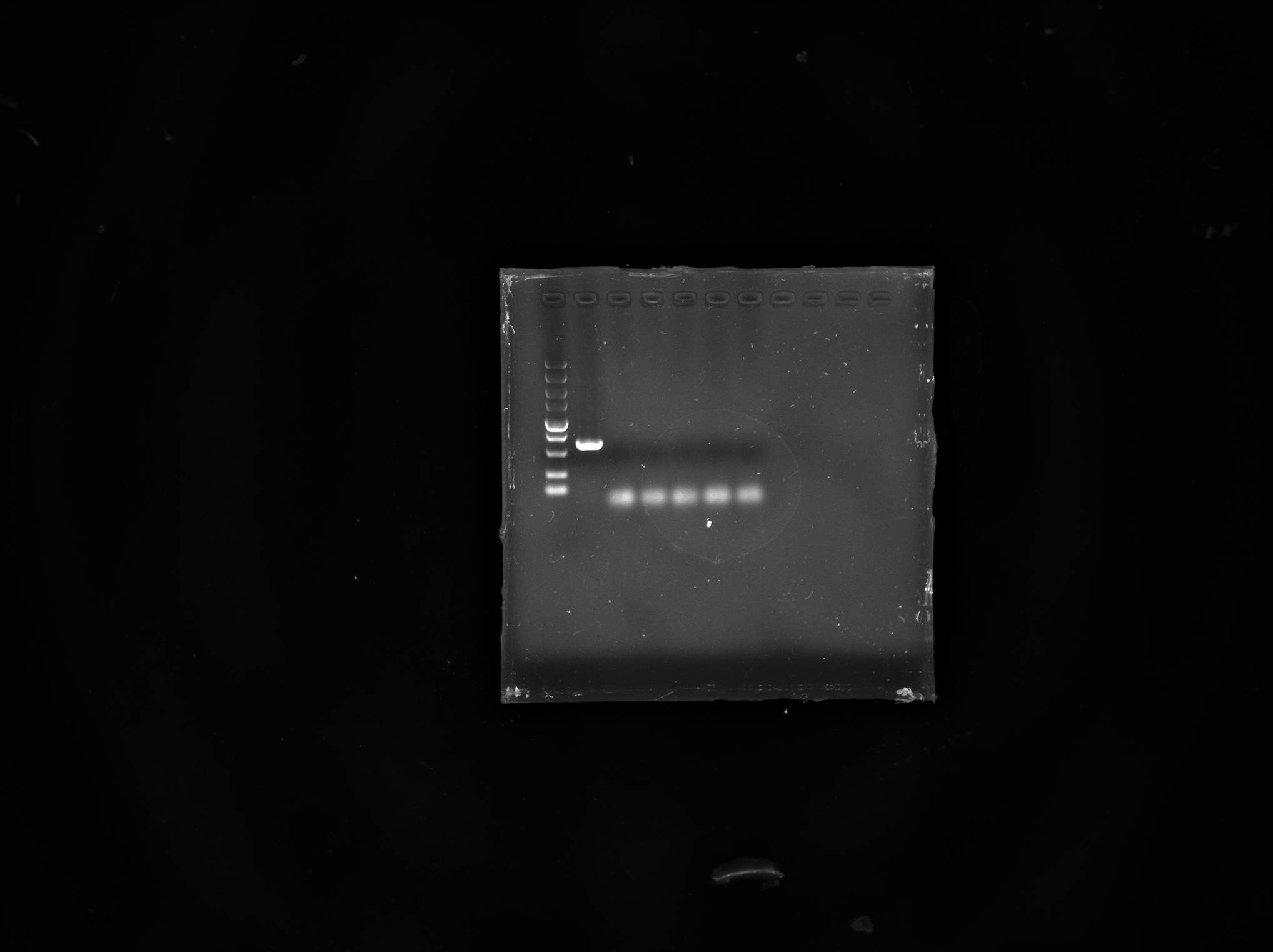

Supplement: Supplementary file 4 [file Image_3.TIF]

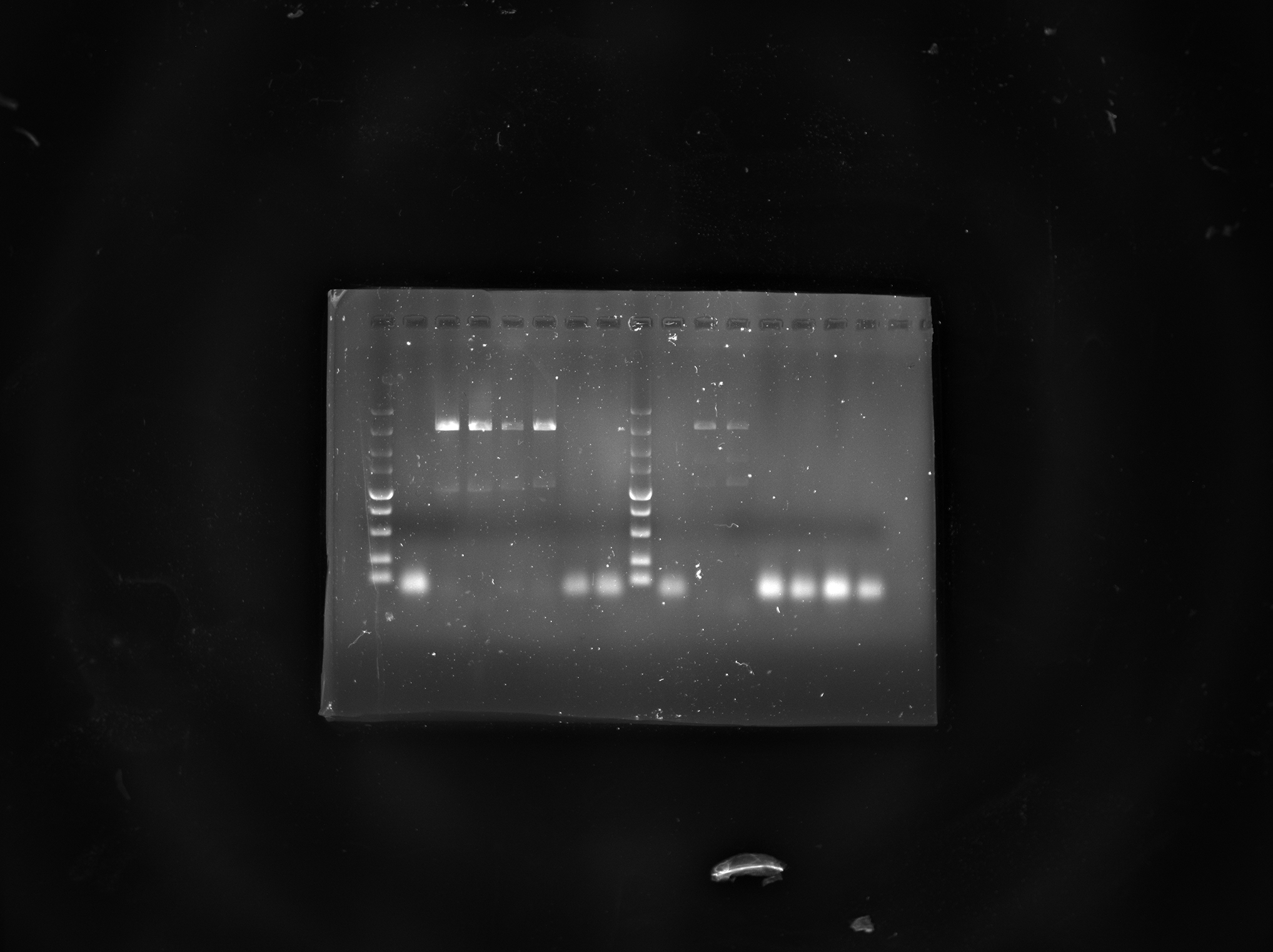

Supplement: Supplementary file 5 [file Image_4.TIF]

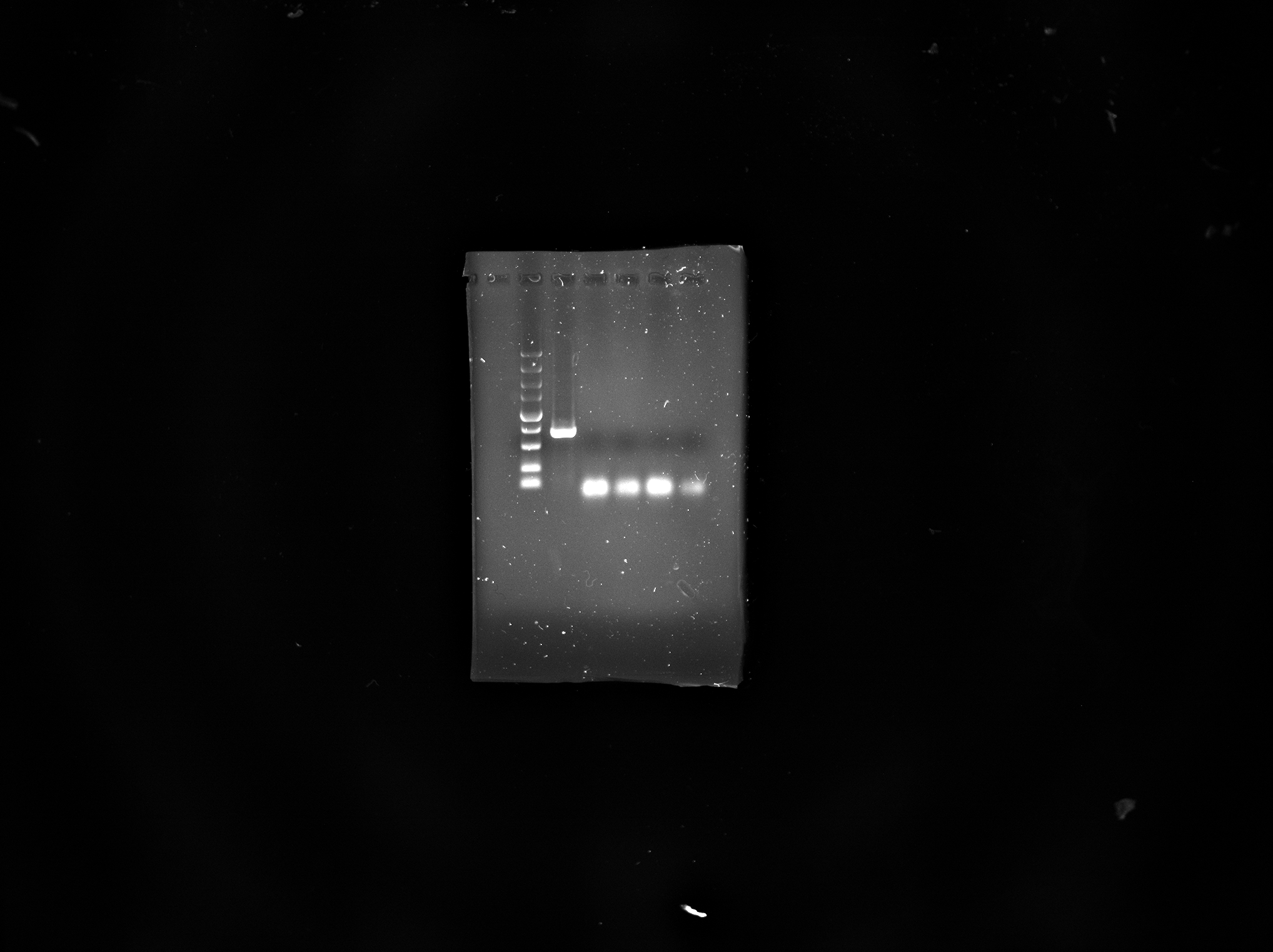

Supplement: Supplementary file 6 [file Image_5.TIF]

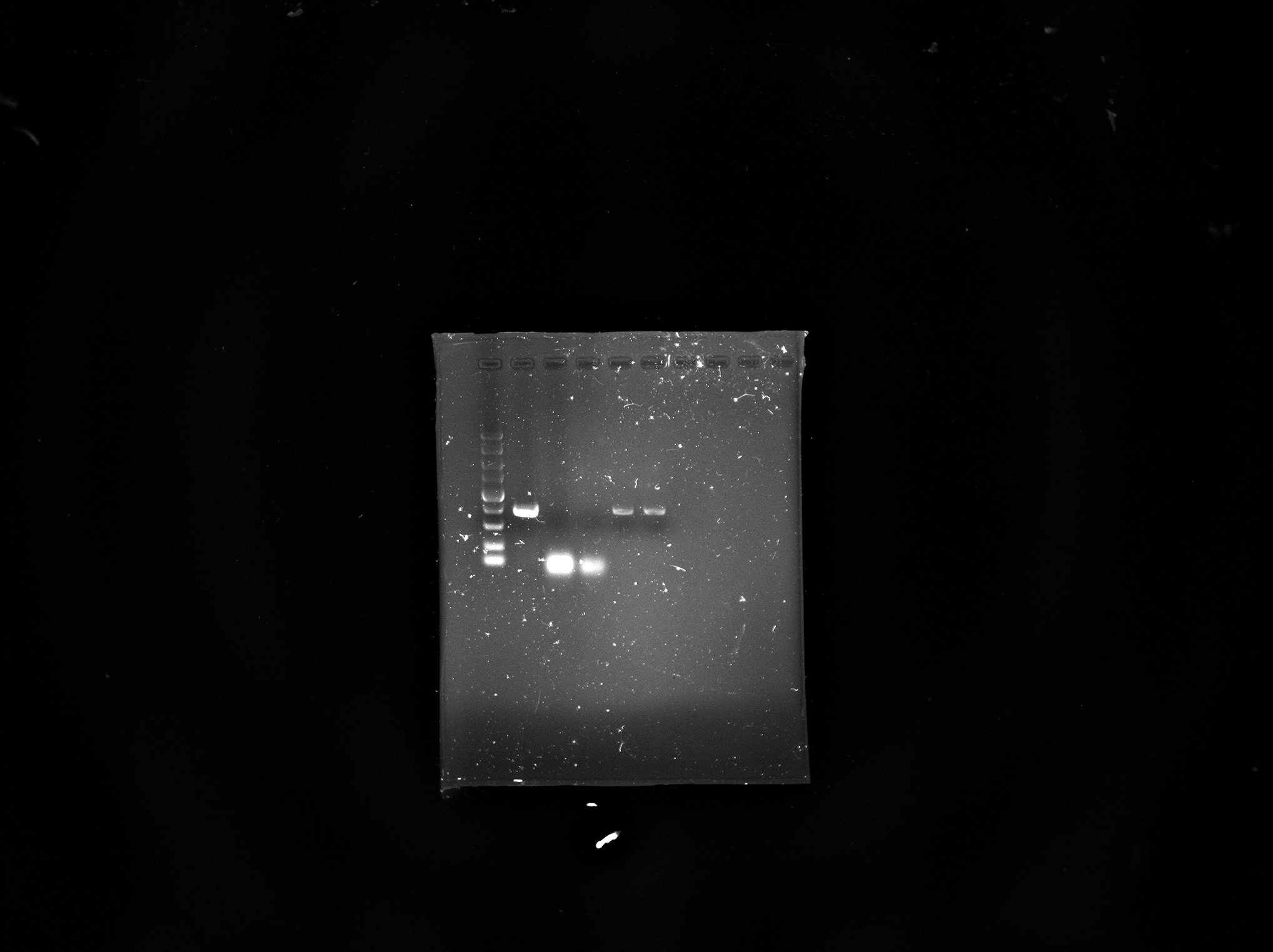

Supplement: Supplementary file 7 [file Image_6.TIF]

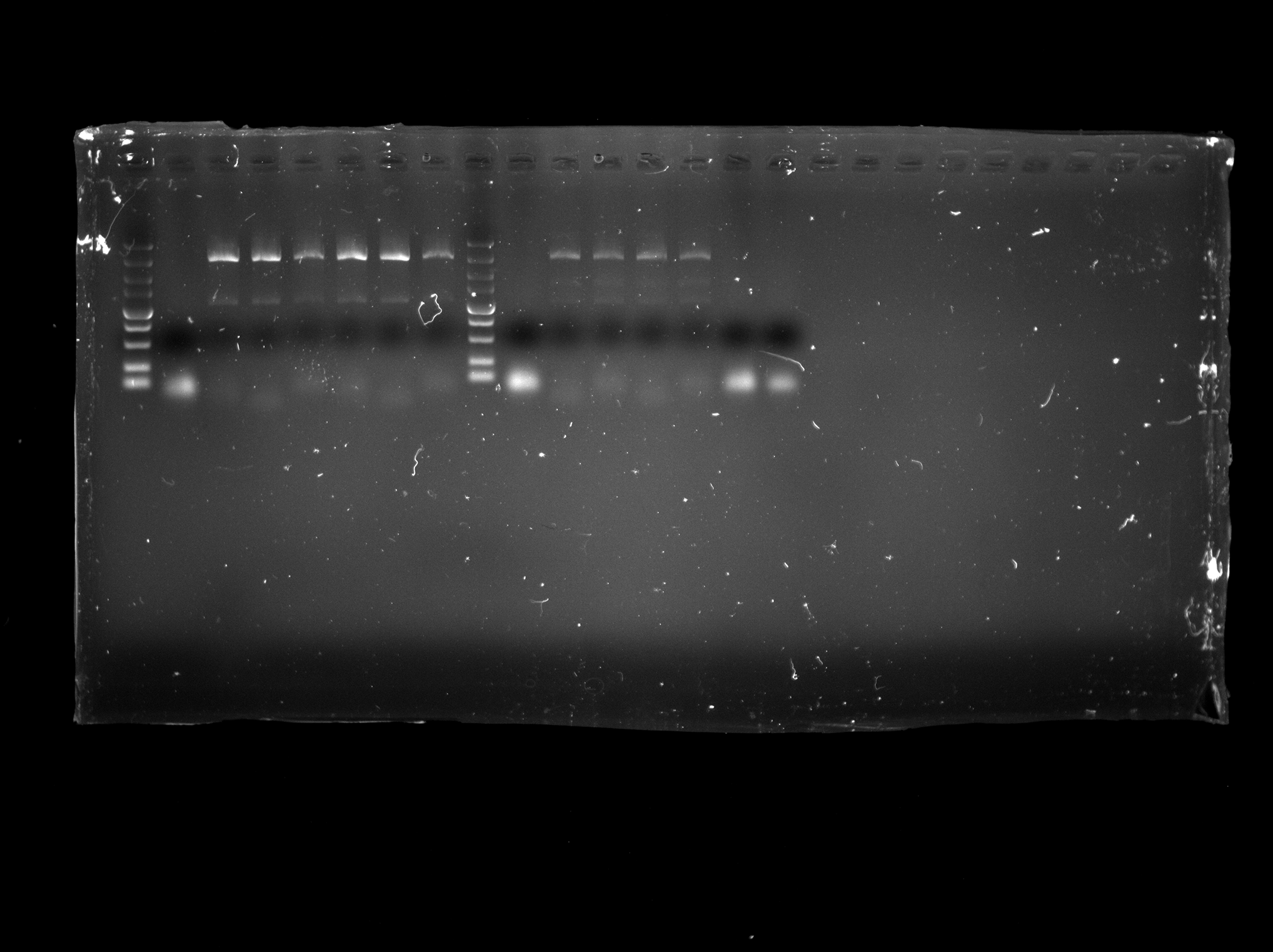

Supplement: Supplementary file 8 [file Image_7.TIF]

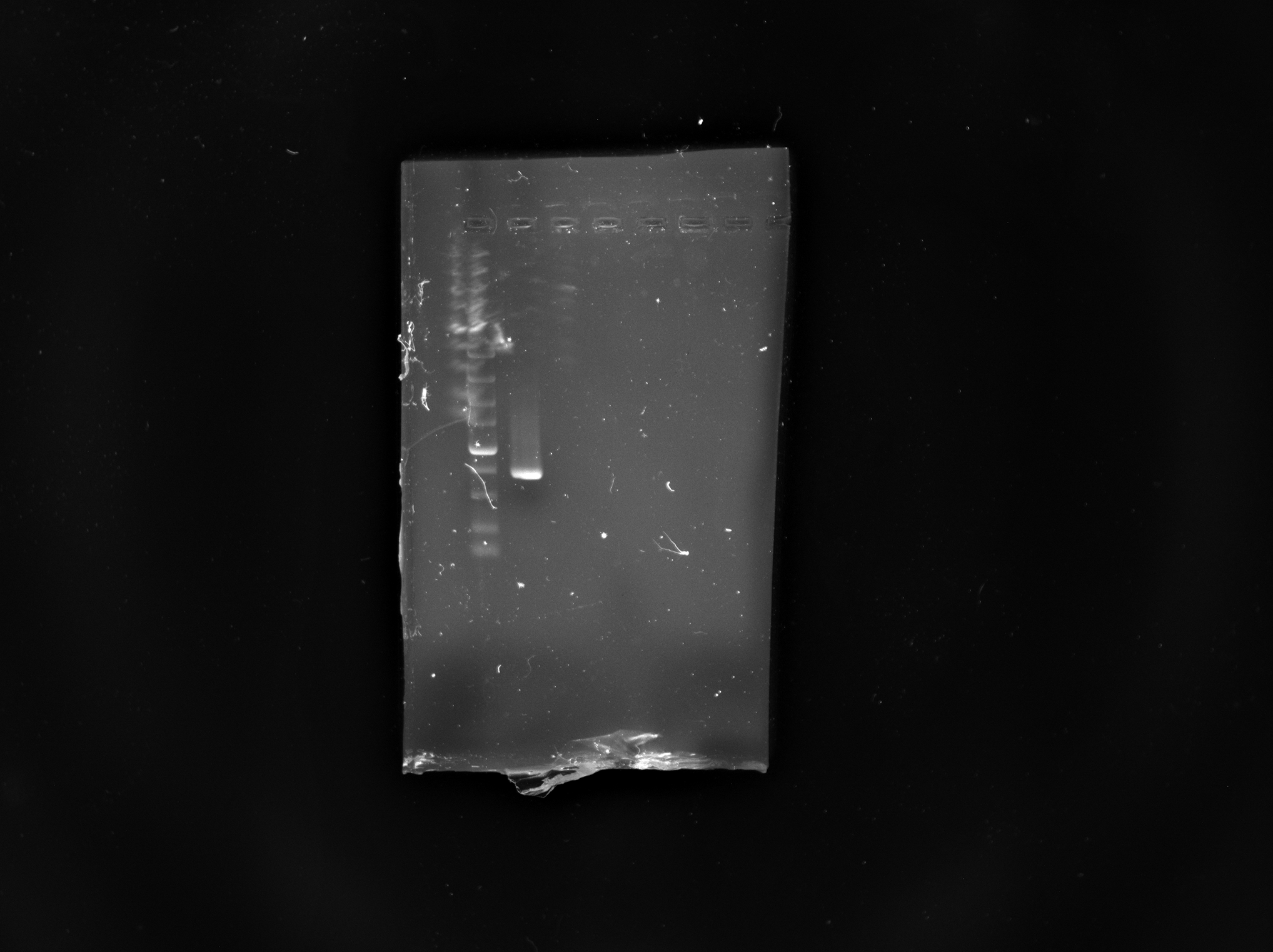

Supplement: Supplementary file 9 [file Image_8.TIF]

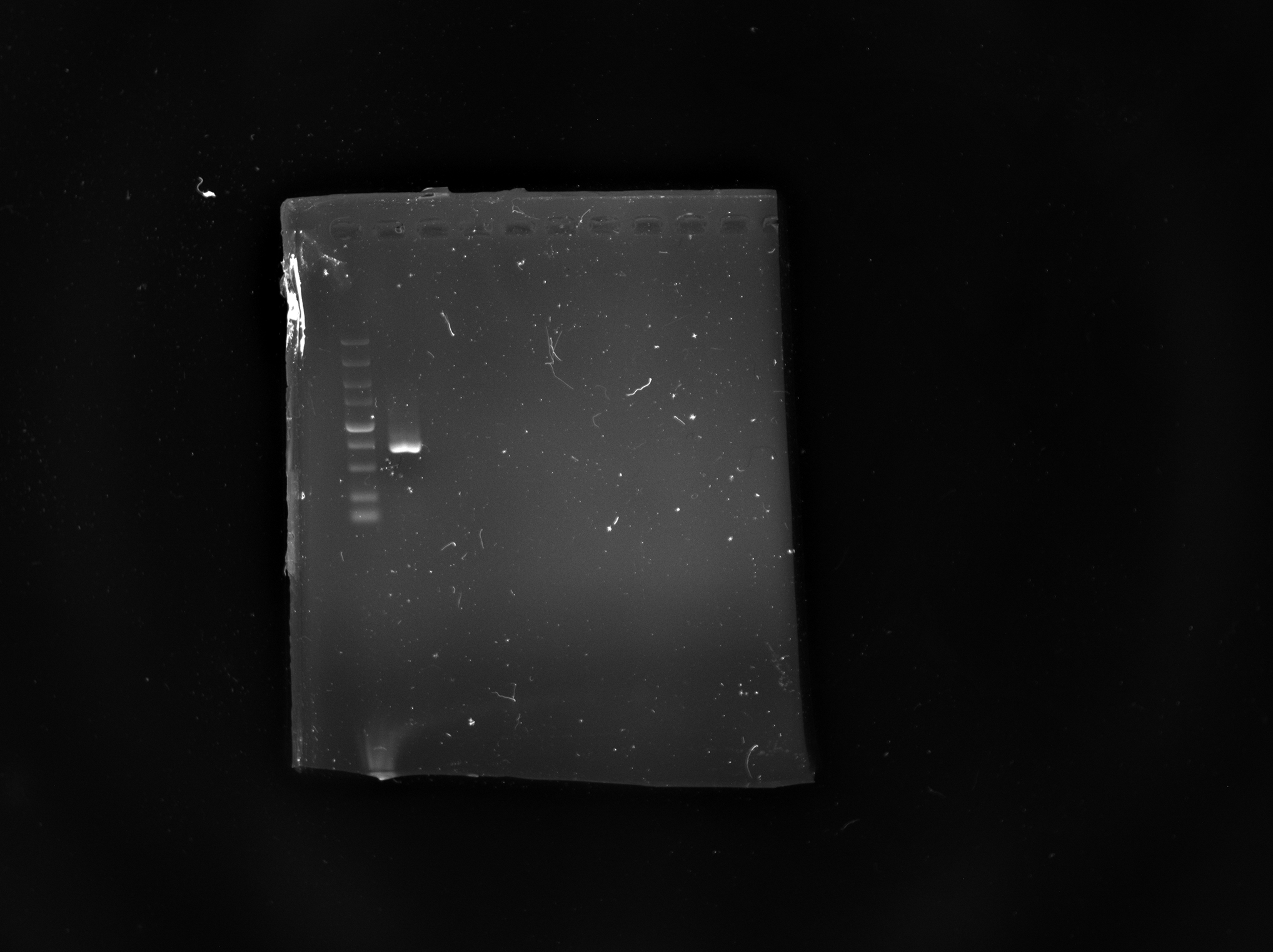

Supplement: Supplementary file 10 [file Image_9.TIF]

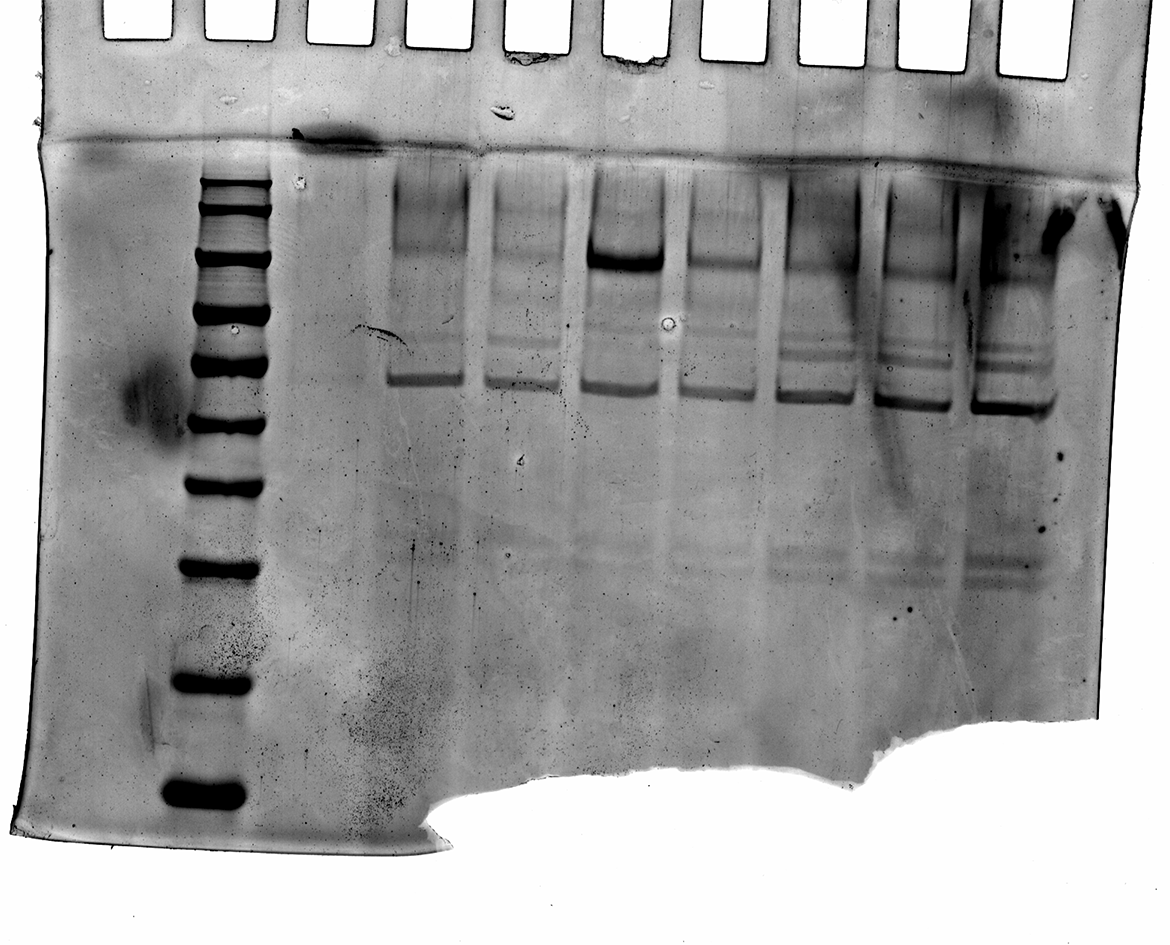

Supplement: Supplementary file 11 [file Image_10.TIF]
